# Low-budget 3D-printed equipment for continuous flow reactions

**DOI:** 10.3762/bjoc.15.50

**Published:** 2019-02-26

**Authors:** Jochen M Neumaier, Amiera Madani, Thomas Klein, Thomas Ziegler

**Affiliations:** 1 Institute of Organic Chemistry, University of Tübingen, Auf der Morgenstelle 18, 72076 Tübingen, Germany

**Keywords:** continuous flow, 3D printing, glycosylation, microreactor, multistep

## Abstract

This article describes the development and manufacturing of lab equipment, which is needed for the use in flow chemistry. We developed a rack of four syringe pumps controlled by one Arduino computer, which can be manufactured with a commonly available 3D printer and readily available parts. Also, we printed various flow reactor cells, which are fully customizable for each individual reaction. With this equipment we performed some multistep glycosylation reactions, where multiple 3D-printed flow reactors were used in series.

## Introduction

The use of flow chemistry in comparison to batch chemistry shows great benefits like better mixing, more efficient heat transfer, and less scale-up problems [1]. For these reasons the number of publications in this field is rapidly increasing over the last decades. Another benefit of flow syntheses is the opportunity to perform multistep reactions with several reactors in a single flow [2–4]. One use of such multiple step reactions is, for instance, the on-demand production of pharmaceuticals using compact, reconfigurable continuous flow systems [5].

The combination of flow chemistry with 3D-printed reactors is also a growing terrain in the last years [6–12]. 3D-printing, also known as additive manufacturing, is a process, where the object is created layer by layer directly from the computer-aided design (CAD) model. There are different technologies available for printing continuous flow reaction devices like fused deposition modeling (FDM) [13–14], selective laser sintering (SLS) [15], or stereolithography (SLA) [16–17]. Each method however, has advantages and disadvantages [18]. While the printing with SLA and SLS allows a very high resolution, the used photopolymer materials in stereolithography printing are poorly resistant against standard organic solvents and in powder-based printing (i.e., SLS) the unsintered powder remains in the channels, which could lead to plugging of the printed device [19]. On the other hand, fused deposition modeling is an inexpensive technology and, especially when the reactor is printed in polypropylene (PP), it shows a good resistance towards common solvents. A disadvantage of FDM is the relatively low resolution [20].

Oligosaccharides, especially as glycoconjugates, play a crucial biological role in nature, e.g., for signal transduction in cell–cell recognition, infection processes, and immunology [21–22]. There are some examples of glycosylation reactions under flow conditions in the literature, which gave promising results so far [23–26]. Therefore, we used custom designed 3D-printed reactors to perform various glycosylation reactions under flow conditions also for demonstrating the applicability of our flow system for such reactions. For studying biological interactions of saccharides and glycoconjugates it is crucial to chemically synthesize such compounds since material isolated from natural sources is often insufficiently pure. In such syntheses, the glycosylation step is usually the most crucial one and it would be desirable to apply flow chemistry to this endeavour.

## Results and Discussion

### 3D-Printed flow reactors

For our reactors, we chose a low-budget FDM 3D printer (Anet A8) which was custom-modified to improve the printing quality. The main advantage of FDM printed reactors in organic synthesis is the use of the inexpensive and chemically robust material PP. There are several examples for 3D printed devices, like microfluidic flow reactors with 50 µm channel width or complex mixing chambers, manufactured with SLA technology [6,12,19], but one significant disadvantage of such systems is the low chemical resistance of the used printing materials towards most organic solvents [11].

**Figure 1:**
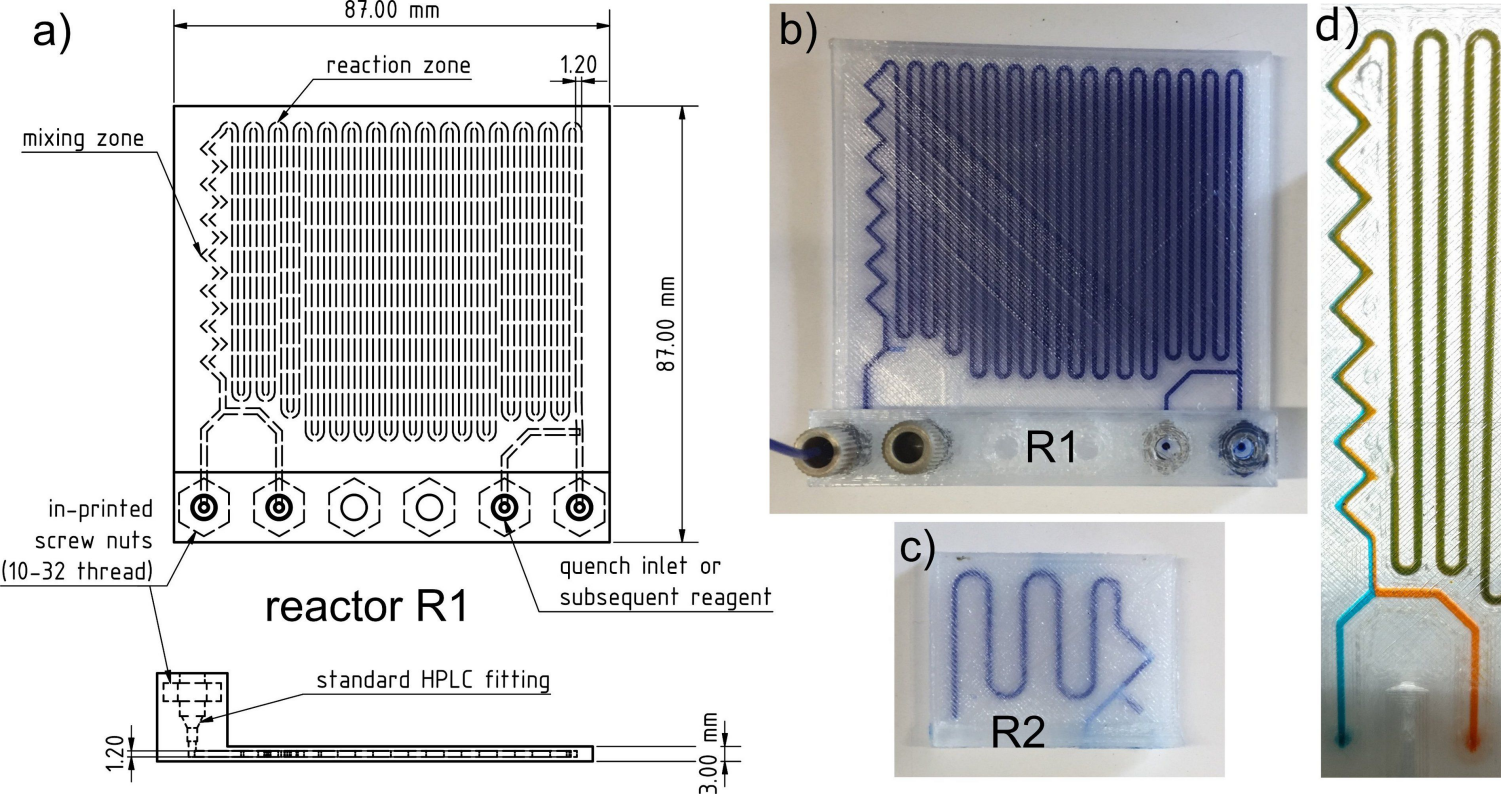
a) CAD drawing of the reactor R1. b) 3D-printed reactor R1 from the CAD drawing. The reactor is filled with a blue dye solution for better visibility of the channels. c) Early stage prototype of a 3D-printed flow reactor (R2). d) Mixing test of two dye solutions.

For the development and construction of our flow reactor we had to print and design numerous devices in order to find the most suitable parameters. All reactors were printed with a filament flow of 105–110% to ensure the necessary tightness of the reaction channels of the device. First, we started printing the reactor in a vertical way, orthogonal to the glass bed of the printer. This kind of printing, however, resulted in warping of the PP. Furthermore, the reaction channels were not leak-proof. Therefore, we printed the reactors horizontally, lying flat on the glass bed. With this printing technique we had been able to print leak-proof reactors with only minimal warping. Figure 1 shows an early stage prototype reactor R2 with large distances between the reaction channels. In this reactor the channel profile was circular with a diameter of 1.5 mm as it was described by Cronin et al. [20]. We found that the round channel shape did not reliably lead to leak-proof reactors. Therefore, we used square channel profiles in which the inner walls of the channels always have the same distance and the printing path is always exactly on top of the previous layer. To elongate the reactor path, we decreased the distance between the channels. Though we made sure that, during the slicing process, the space between the channels is not printed with the infill function of the slicing software, but with continuous lines. This was guaranteed by sizing the wall thickness to a multiple of the nozzle diameter. With a standard nozzle diameter of 0.4 mm, a resulting wall thickness of 1.2 mm (3 × nozzle diameter) led to the reliably leak-proof reactor R1, in which most of the glycosylation reactions were performed. The shape of the channels is a square with 1.2 mm × 1.2 mm diameter and the reactor has a total volume of 1.05 mL. It was designed to have two initial inlets with a subsequent chaotic mixing zone [27] and one quench inlet. We chose two dye solutions in dichloromethane for mixing experiments as a model for our reactions to estimate the mixing efficiency (Figure 1d). The simple zigzag shaped mixing channel is a good compromise between easy printability and sufficient mixing performance. Breadmore et al. also showed that FDM printed reactors innately provide a better mixing quality in laminar flow due to the inner roughness of the channels [28].

Our design allows for a flexible change via CAD software in a few minutes. For instance, adding more inlet ports or altering the channel dimensions. The modified reactor can be printed in a few hours.

**Figure 2:**
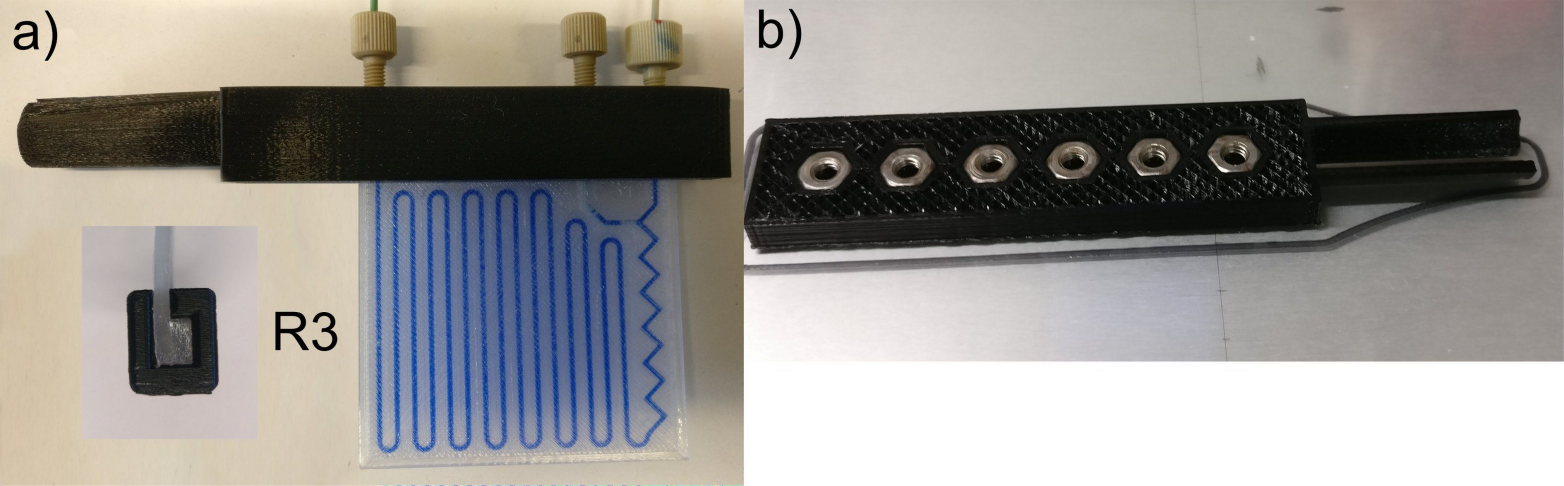
a) L-shaped rail made of PLA with the mounted reactor R3. The small picture shows the fixed reactor in the rail. b) In-printed screw nuts during 3D-printing: The print was paused, the nuts inserted and the print resumed.

We also optimized the way how to establish a safe and reliable connection from the reactor to the tubing. For that purpose, we used a 1/16 inch ethylene tetrafluoroethylene (ETFE) tubing (0.75 mm ID) with standard PEEK HPLC fittings (10–32 thread). Due to the soft mechanical properties of PP, it is very difficult to cut a thread into the reactor after printing. Therefore, we printed L-shaped rails out of polylactic acid (PLA) containing manually cut threads or in-printed screw-nuts (Figure 2). The best and most reproducible method was to in-print the screw nuts into the reactor itself (see Figure 1a).

**Figure 3:**
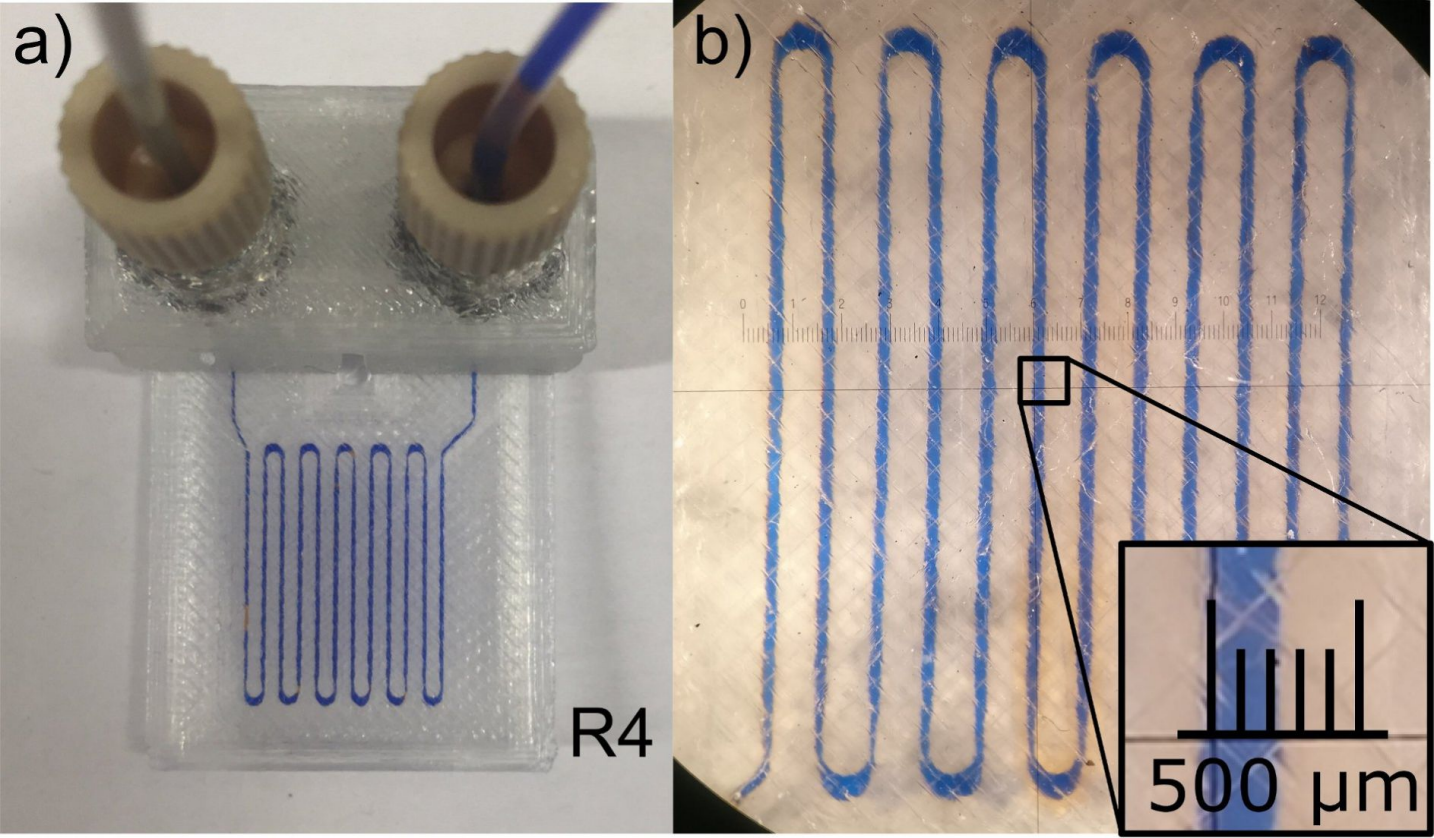
a) Microreactor R4 with a reactor volume of 12 µL filled with a blue dye solution. b) Magnification of the reactor channels, which shows a width of ≈200 μm.

In order to determine the limitation of the channel resolution for our printing method, we designed test reactors with a very small channel width down to 0.1 mm. Here, the smallest possible resolution appeared to be a profile area of 0.3 mm × 0.3 mm in the CAD drawing which resulted in channel width of around 0.2 mm (Figure 3). Due to polymer spreading during extrusion, we usually obtained channels about 100 µm smaller than set in the CAD drawing. The same effect was previously also encountered by others [28]. A smaller channel resolution than 200 µm is nearly infeasible because it repeatedly led to a blockage of the channels as previously observed by others as well [29].

Although 3D printing of reaction devices is a growing field in the last years, there are only a few examples of organic reactions with FDM-printed reactors [7]. Reactors made of materials like PLA, ABS (acrylonitrile butadiene styrene) or HIPS (high-impact polystyrene) are described in the literature [29–30], but these materials are limited to a small range of reaction conditions and so not comparable to our reactors. For our purposes, a printing material is needed which is resistant to a wide range of reaction conditions, like PP. Besides us, only a few groups, like Cronin et al. and Hilton et al., are also using PP as a printing material [14,20,31]. In comparison our reactors show a very fine structure, for example, as shown in Figure 3, we are able to print microreactors out of PP with a channel width of 200 µm which is to our knowledge the tightest channel achieved in FDM-PP printing.

**Figure 4:**
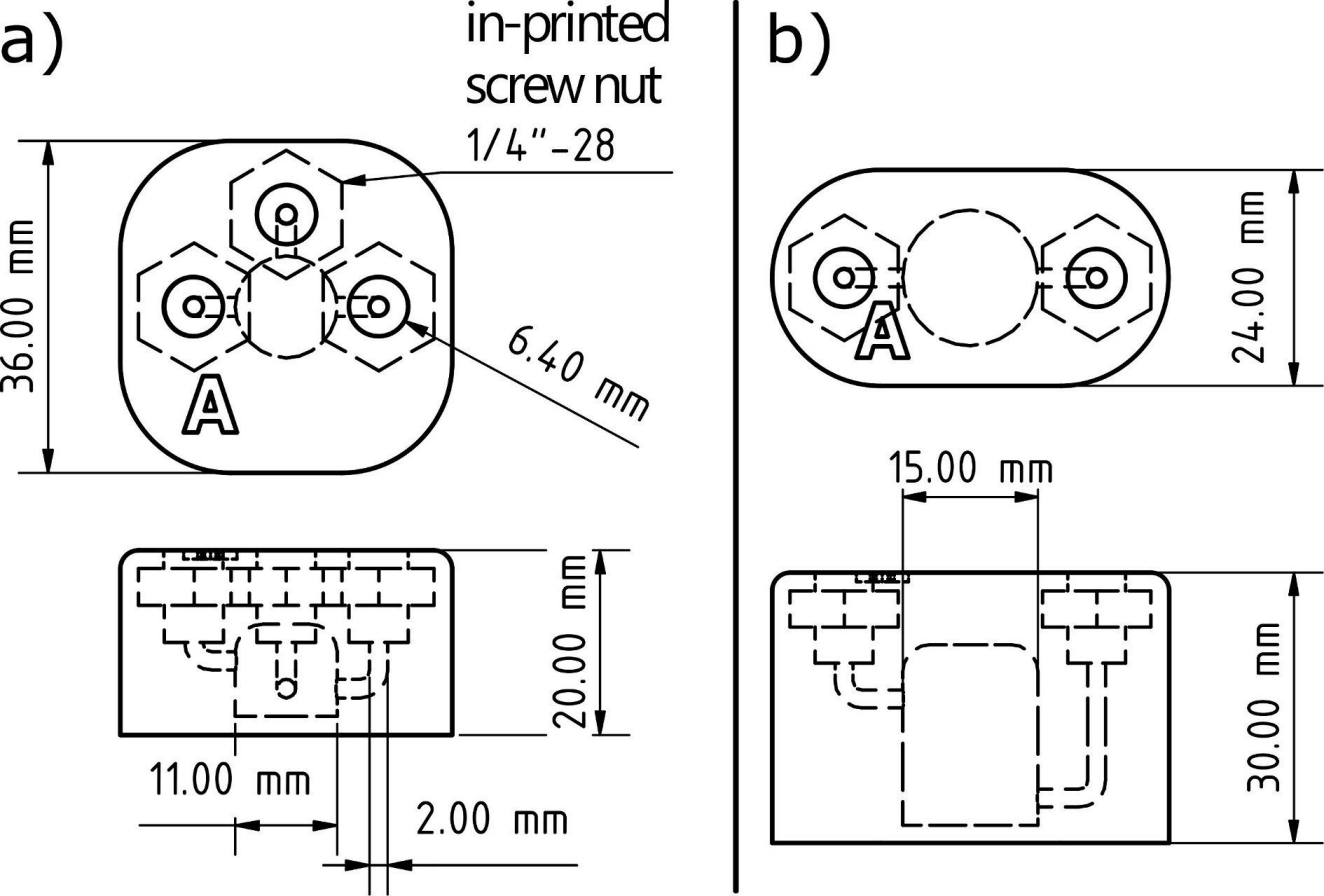
CAD drawing of two CSTR with three (a) and two inlets (b) with in-printed screw nuts 1/4’’ – 28 thread for flat bottom fittings.

Next, we also constructed two types of continuous stirred tank reactors (CSTR) with two and three inlets (Figure 4) essentially following previously published designs [32]. We used 1/4’’ – 28 flat bottom fittings for our reactors. Thus, either 1/16’’ or 1/8’’ tubing with a larger inner diameter could be used if precipitates are formed during a reaction which is often a problem in continuous flow reactions [33]. For mechanically mixing, a small magnetic stirring bar was placed in the reactor during printing. These reactors were used for the premixing of reactants or for the extraction steps during the flow syntheses in order to ensure good mixing behaviour of the aqueous and organic phases.

### 3D-Printed syringe pumps

**Figure 5:**
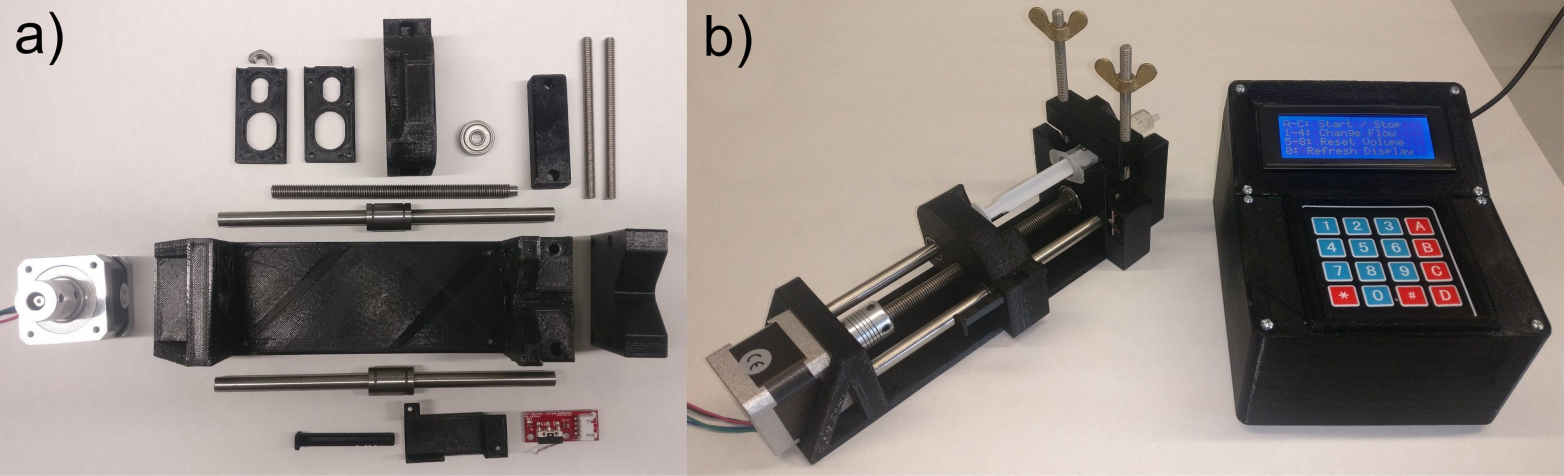
a) Unassembled parts used for one syringe pump. b) Assembled pump with controller.

We further decided to develop and manufacture low-cost and simple-to-use 3D-printed syringe pumps. The materials and parts which were used for a rack of four pumps controlled by one Arduino Mega 2560 did cost less than 300 €. We intended to make the system as simple as possible, so it allows for easy cloning, modification and improvement by others. The frame parts of the pump were 3D-printed out of PLA and only the stepper motors, bearings and all-thread rods, nuts and various screws are necessary for the assembly (Figure 5). For a full part list, CAD files of the printed parts and manufacturing details see the Supporting Information. The control command program was written on the open-source Arduino software and was fully adaptable to syringes from 1 mL to 50 mL. After the first pump tests, we found the accuracy of the dispensed volume to be insufficient. Therefore, each syringe was calibrated individually resulting in deviations below 1%.

### Glycosylation reactions

**Scheme 1:**
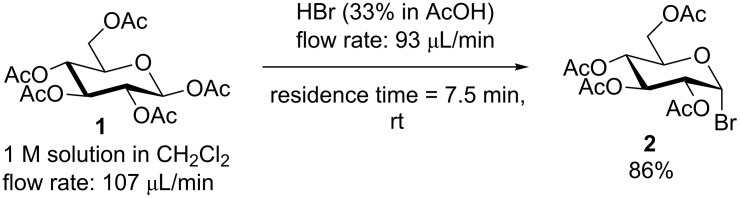
Preparation of acetobromo-α-D-glucose **2**.

With our custom made 3D-printed reactors and pumps in hand, we performed some simple glycosylation reactions as a proof of concept for our hardware and for reactor setup. We first started with the optimization of the synthesis of the commonly used glycosyl donor acetobromo-α-D-glucose **2** under flow conditions. To the best of our knowledge, the preparation of glycosyl bromides under continuous flow conditions has not been published so far. For the synthesis of acetobromo glucose we used the reactor R3 (Figure 2) with a total volume of 1.5 mL. We first studied various solvents, reaction times, equivalents of HBr and temperatures for the optimization of the reaction. The best conditions are shown in Scheme 1. Faster flow rates (higher than 200 μL/min) led to incomplete conversions of the starting material. A flow rate resulting in a residence time of 7.5 min enabled a production rate of ≈5 g/h with a yield of 86%, due to the high concentration of the starting materials.

**Figure 6:**
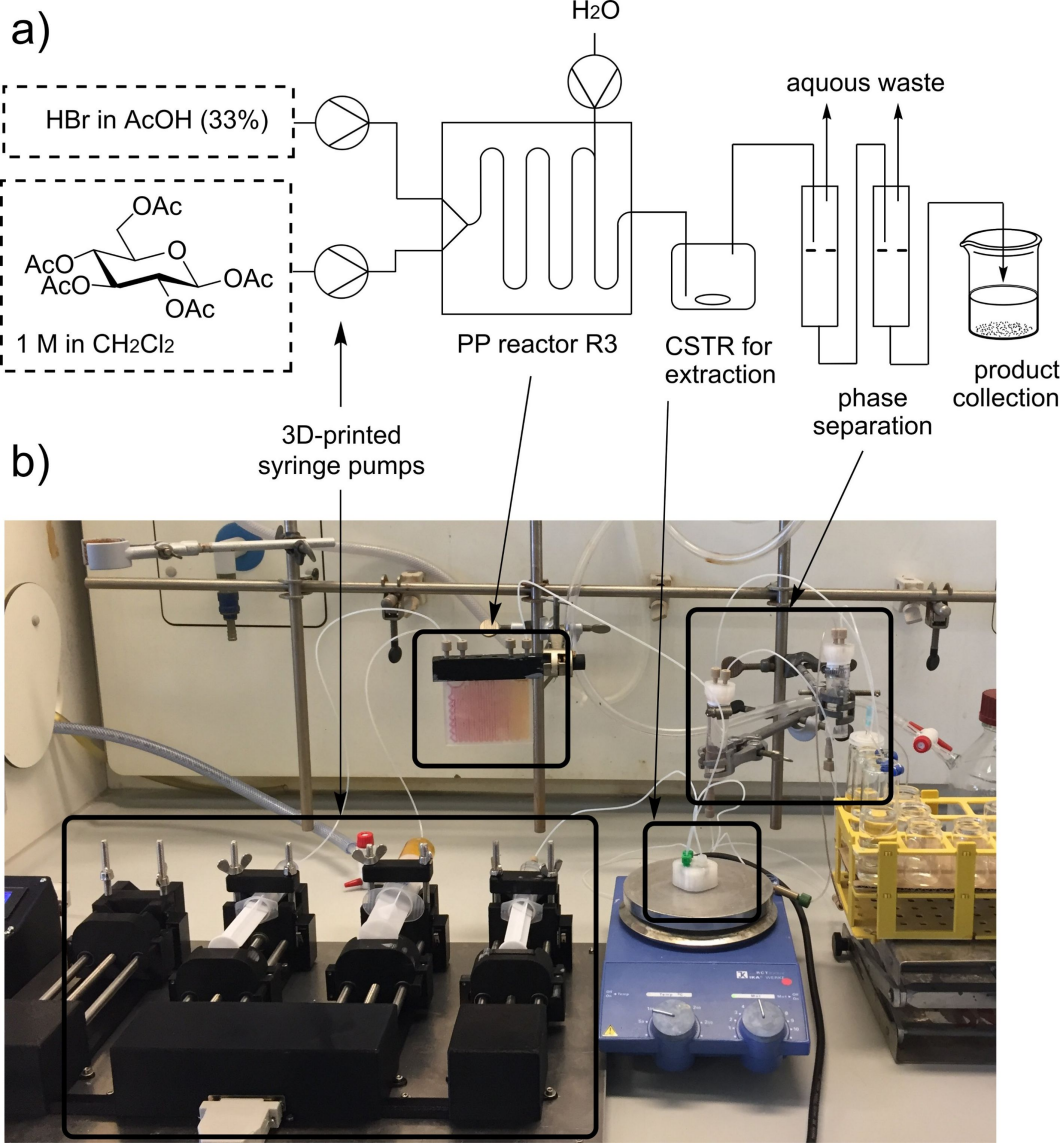
a) Schematic diagram for the continuous-flow synthesis of acetobromo-α-D-glucose **2**. b) Photograph of a typical setting for continuous flow reaction.

In Figure 6 the setup and a photograph of the reaction with the reactor, the CSTR for extraction and a 10 mL syringe for phase separation is shown. It consists of two 3D-printed pumps for solutions of pentaacetylglucose in dichloromethane (1 M) and HBr in acetic acid (33%) and PP reactor R3. It should be noted that the reactor material withstood the harsh acidic conditions. Work-up of the reaction mixture and isolation of acetobromo glucose **2** was done by passing the reaction mixture through a CSTR device and two syringes for phase separation [34–35]. The procedure can easily get scaled up and provides for a convenient method for preparing acetobromo glycoses.

**Scheme 2:**
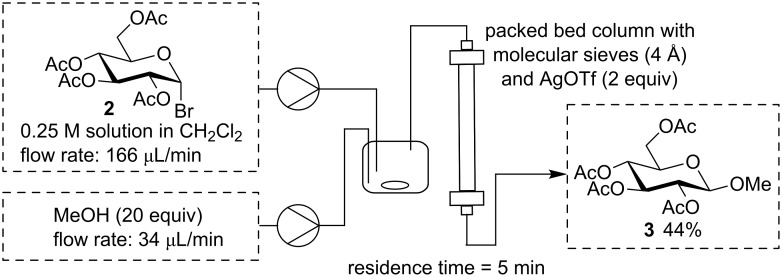
Flow Koenigs–Knorr reaction to methyl glycoside **3** with silver triflate.

In order to show the suitability of our flow system for the preparation of simple glycosides, we first tested Koenigs–Knorr glycosylation conditions with silver triflate as activator. Thus, silver triflate (2 equiv) was mixed with molecular sieves (4 Å) and placed in a packed bed reactor. Next, a solution of acetobromo glucose (**2**) in dichloromethane (0.25 M) and methanol were pumped through the reactor at such a rate that ca. 20 mol equivalents of methanol were present in the mixture. Scheme 2 shows the setup of the glycosylation reaction under flow conditions. Best results for the synthesis of methyl glycoside **3** were obtained using an overall flow rate of 200 μL/min leading to a residence time of 5 min and a yield of 44%. A similar Koenigs–Knorr type glycosylation under continuous flow has only been described previously for glucuronidation of bile acids [36].

**Scheme 3:**
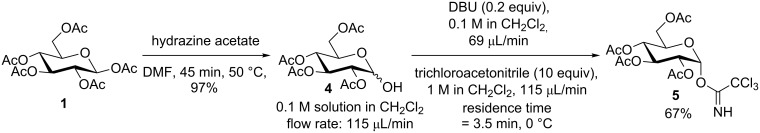
Preparation of glycosyl donor **5**.

Unfortunately, the synthetic steps for the glycoside preparation could not be combined in a multistep reaction, due to clogging of the packed bed reactor, most likely due to the formation of silver bromide during the Koenigs–Knorr reaction. No such clogging was observed when the column was packed with Fetizon’s reagent (Ag_2_CO_3_ on Celite) [36–37]. The bromination and glycosylation reaction steps had to be performed in separate reactions. Therefore, we also investigated glycosylations with the respective imidate glycosyl donor **5**. First, the cleavage of the anomeric acetyl group with hydrazine acetate was performed under batch conditions according to the literature [38] (Scheme 3). The following conversion of glucose **4** into trichloroacetimidate **5** was done in a flow reactor type R1 with a reaction time of 3.5 min. This way, a yield of 67% was obtained for this glycosylation step. Once again we found that longer residence time resulted in a lower yield (10.5 min = 37%). Similar observations were previously made for glycosylation reaction under continuous flow conditions [23–26]. A larger amount of DBU did not increase the yield.

**Scheme 4:**
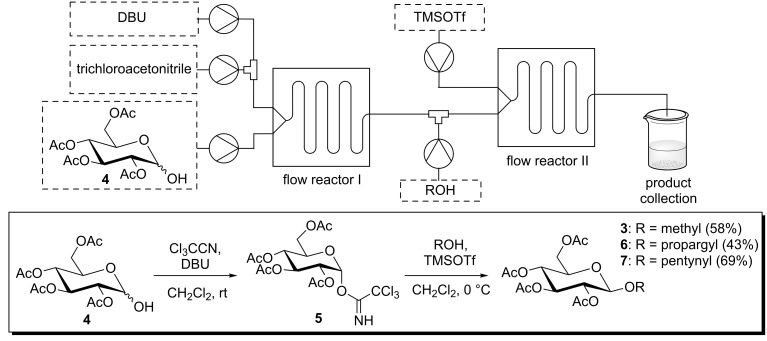
Two-step glycosylation reactions starting from pyranose **3**.

For the glycosylation reactions with glycosyl donor **5** a multiple step reaction arrangement starting from pyranose **4** was set up. Scheme 4 shows this setup of a suitable cascade reaction in which DBU, trichloroacetonitrile and pyranose **4** were pumped through the first flow reactor (R1) followed by the addition of the alcohol and TMSOTf through the second reactor. Table 1 shows the detailed reaction conditions for methanol, propargyl alcohol and 4-pentynol.

**Table 1:** Reaction conditions for the two-step glycosylation.

product	reagents	flow rate [μL/L]	molarity [mol/L]	equiv	residence time [min]	isolated yield

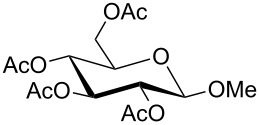 **3**	pyranose **4**	67	0.1	1	7.5	58%
	trichloroacetonitrile	67	1	10		
	DBU	13	0.1	0.2		

	methanol	133	0.1	20	3.5	
	TMSOTf	20	0.01	0.3		

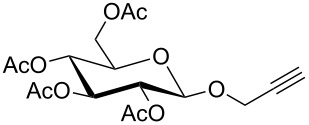 **6**	pyranose **4**	56	0.1	1	7.5	43%
	trichloroacetonitrile	56	1	10		
	DBU	28	0.1	0.5		

	propargyl alcohol	56	1	10	4.2	
	TMSOTf	56	0.1	0.7		

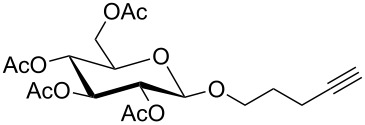 **7**	same reaction conditions as for glycoside **6**, with 4-pentynol as alcohol					69%

The two-step reaction starting from pyranose **4** gave overall yields in the range from 43% to 69%. Due to the neighbouring group participation of the acetyl group at C-2, only β-anomers of the respective glycosides **3–7** were obtained. To the best of our knowledge similar cascade flow glycosylations have not been described in the literature so far.

**Scheme 5:**
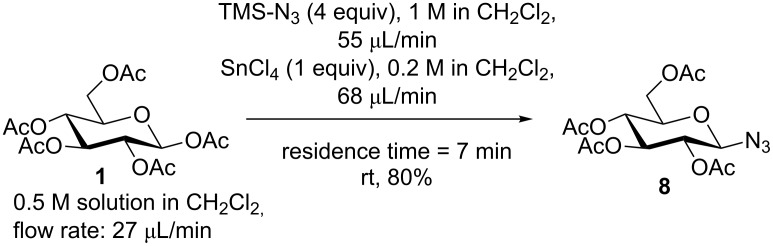
Synthesis of azide-functionalized glycopyranoside **8**.

Finally, the herein developed flow system devices could also be applied to the continuous flow preparation of glycosyl azides. For example, we were able to convert pentaacetyl glucose (**1**) with trimethylsilyl azide in the presence of SnCl_4_ directly into azide **8** (Scheme 5) as was previously described for the classical batch preparation [39]. At a resident time of 7 minutes an 80% yield of azide **8** could be achieved.

## Conclusion

In conclusion, we have demonstrated that low-budget lab equipment for continuous flow chemistry could be manufactured for under 300 €. With this equipment, consisting of Arduino controlled syringe pumps and microreactors, the preparation of glycosyl donors and glycosylation reactions were performed in a cascade fashion to show the viability of this system.

## Supporting Information

File 1 All details for the 3D-printed lab equipment and reactors (full part list, exploded-view CAD drawings, Arduino wiring) and all experimental data of the chemical reactions and NMR spectra.

File 2 This zip-file includes all 3D-printed parts as stl-files for direct 3D printing, as well as stp-files for editing the 3D models, if necessary. It also contains the Arduino software code as an ino-file for controlling of the syringe pumps.
