# Supplementary material for: Low-budget 3D-printed equipment for continuous flow reactions
Source: Beilstein J Org Chem. 2019 Feb 26;15:558–66. doi: 10.3762/bjoc.15.50 (PMC6404462; doi:10.3762/bjoc.15.50)
Supplement: File 1 — All details for the 3D-printed lab equipment and reactors (full part list, exploded-view CAD drawings, Arduino wiring) and all experimental data of the chemical reactions and NMR spectra. [file Beilstein_J_Org_Chem-15-558-s001.pdf]

BEILSTEIN JOURNAL OF ORGANIC CHEMISTRY

Supporting Information

for

Low-budget 3D-printed equipment for continuous flow reactions

Jochen M. Neumaier, Amiera Madani, Thomas Klein and Thomas Ziegler

Beilstein J. Org. Chem. **2019,** *15,* 558–566. doi:10.3762/bjoc.15.50

All details for the 3D-printed lab equipment and reactors (full part list, exploded-view CAD drawings, Arduino wiring) and all experimental data of the chemical reactions and NMR spectra

License and Terms: This is a supporting information file under the terms of the Creative Commons Attribution License (http://creativecommons.org/licenses/by/4.0). Please note that the reuse, redistribution and reproduction in particular requires that the authors and source are credited.
The license is subject to the *Beilstein Journal of Organic Chemistry* terms and conditions: (https://www.beilstein-journals.org/bjoc)

Contents

Printer modifications S1
3D-printed lab equipment S2
General information S9
Experimental S10
NMR spectra S16
References S30

Printer modifications

The 3D Printer is an A8 from Anet, which has been modified to improve the print results and to allow a stable, safe and convenient operation. For safety reasons the power supply unit has been equipped with active ventilation (1), a power switch (2) and a fuse (3). Instead of the original heating cartridge, a custom-made device from HS Heizelemente has been installed (4). All 12 V and 230 V wires were upgraded with proper connections and the mainboard got two MOSFETs (hotbed and hotend) (5).

Components which helped to improve quality of the prints are for example self-printed *x*-,*y*- and *z*-axis stabilizers (6), *x*- and *y*-axis belt tensioners (7) (all downloaded from Thingiverse.com), a LJC18A3-H-Z/BX capacitive autolevel sensor (8) and a rubber mat under the printer (9).

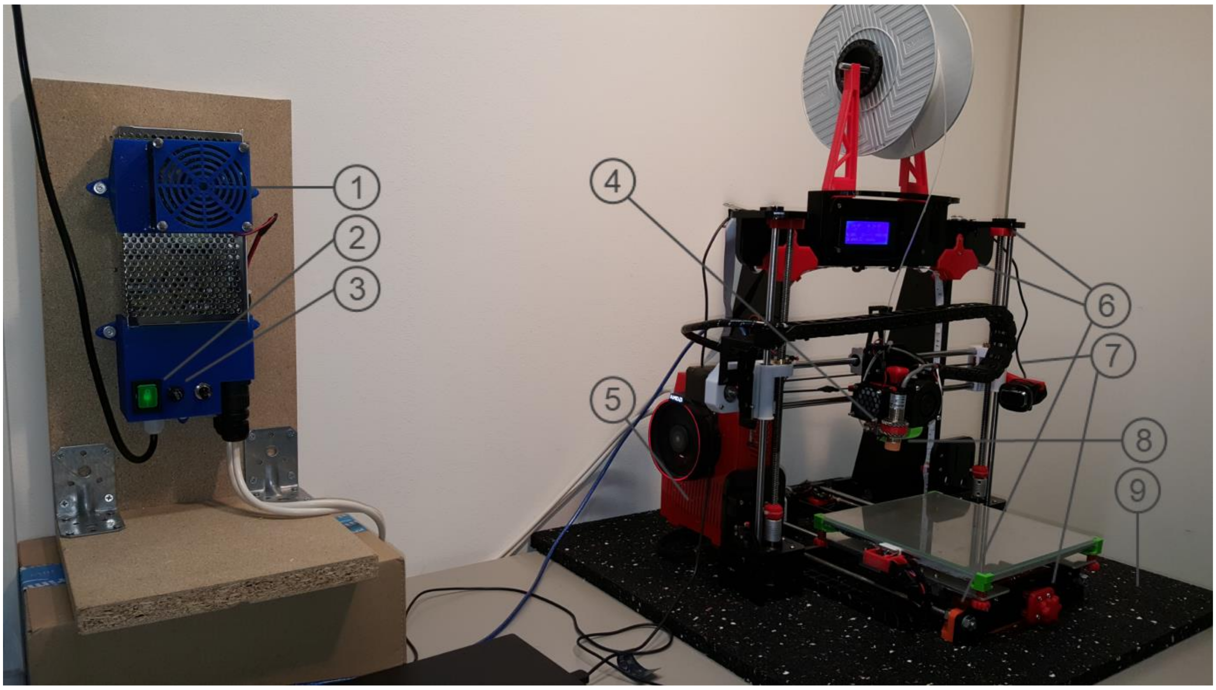

Figure S1: Custom modified 3D Printer Anet A8.

3D-printed lab equipment

Software: The lab equipment and flow reactors used in this work were designed with Autodesk Inventor Professional 2018. After modelling, the files were exported as stl files and sliced with the freeware program Cura 2.7.0 from Ultimaker.

Flow reactors: The reactors were printed with Verbatim PP filament 1.75 mm - Natural Transparent. The best results were obtained using the following settings:

Print speed = 30 mm/s

Filament flow = 105%

Nozzle temp. = 200 °C

Bed temperature = room temperature/no heating, printed on transparent PP packaging tape from Lyreco with a brim of 10 mm

Layer height: 0.1 mm – 0.15 mm (0.1 mm for channel diameter < 1 mm)

Wall thickness: 2.0 mm = 5 × nozzle diameter (0.4 mm)

Fan speed: 100% beginning at layer 3

Syringe pump, 3D-printed parts: The parts for the syringe pump were printed with polylactic actid (PLA) from Janbex. The best results for PLA were obtained using following settings:

Print speed = 60 mm/s

Filament flow = 100%

Nozzle temp. = 200 °C

Bed temperature = 60 °C, printed on a glass plate, coated with hairspray before each print.

Layer height: 0.2 mm – 0.3 mm

The STP and STL files of all printed parts can be found in the zip- file. Figure 2 shows the unassembled and assembled syringe pump as well as exploded-view CAD drawings of the pump and the controller. The part numbers can be found in Table 1.

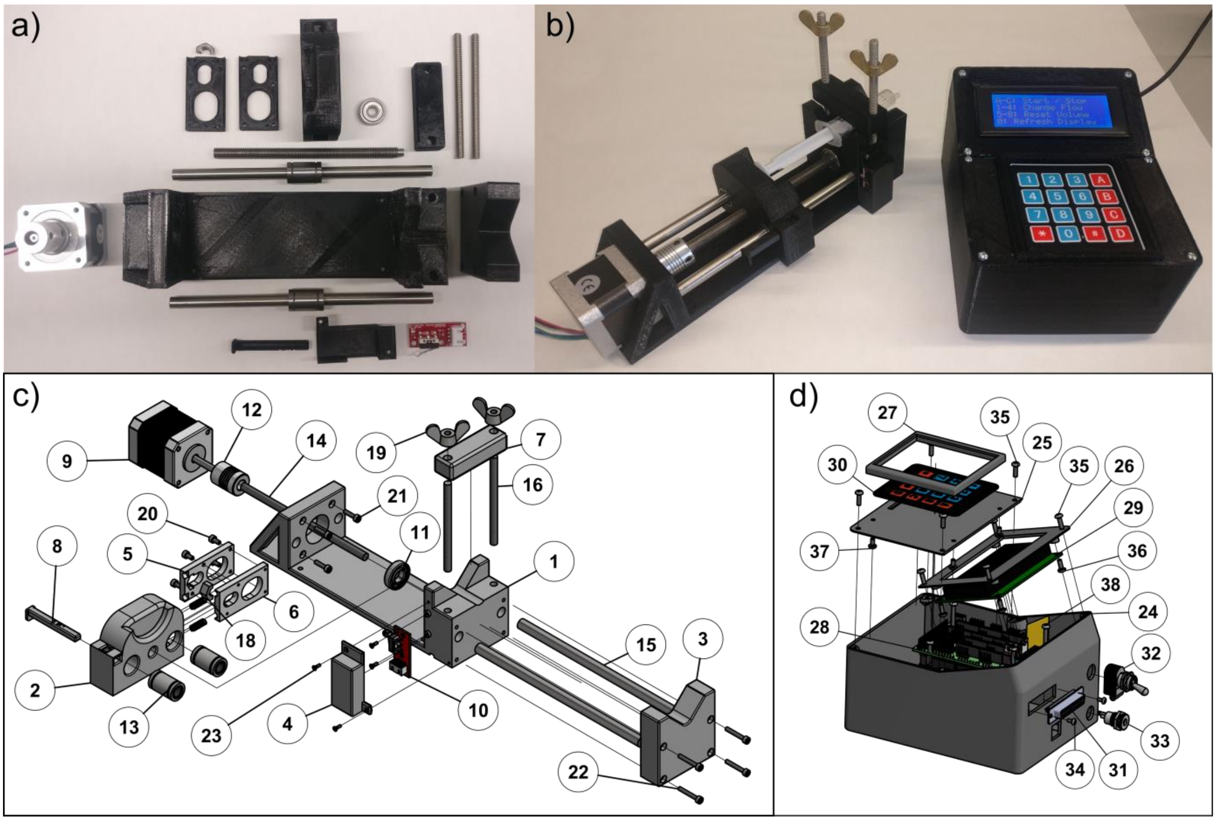

Figure S2: a) Unassembled parts used for one syringe pump. b) Assembled syringe pump and controller c) Exploded-view CAD drawings of the pump. d) The controller.

The "Adjustable end position" part (8) can be set to four positions for the six syringes used in our laboratory. Table 2 shows the right position for each syringe. If other syringes are used, their end-stop positions have to be set manually.

Table S1: Part list for a rack of four pumps with Arduino controller

**Quant.**	**Part**	**Purchased from**	**No.**
	**For the pumps:**		
4	Main body	3D-printed	1
4	Movable component	3D-printed	2
4	Pump ending	3D-printed	3
4	Cap end stop	3D-printed	4
4	Nut holder part 1	3D-printed	5
4	Nut holder part 2	3D-printed	6
4	Syringe holder	3D-printed	7
4	Adjustable end position	3D-printed	8
1	Motor case middle part	3D-printed	
2	Motor case outer part (2 times, mirrored)	3D-printed	
4	Pololu stepper motor NEMA 17, 200 bipolar steps	Eckstein GmbH	9
4	Mechanical limit switch end stop	Eckstein GmbH	10
4	Ball bearing L626ZZ	In-house workshop	11
4	5 mm x 5 mm CNC stepper motor top narrow shaft couplings	Eckstein GmbH	12
4	LM8UU linear bearings	Eckstein GmbH	13
4	Threaded rod, M8 × 135 mm	In-house workshop	14
8	Round rod, 180 × 8 mm	In-house workshop	15
8	Threaded rod, M6 × 80 mm	In-house workshop	16
8	Spring 10 mm, 5 mm diameter	In-house workshop	17
4	Screw nut, M8, half cutted	In-house workshop	18
8	Wing nut, M6	In-house workshop	19
16	Cheese head screw with hexagon socket, M3 ×16 mm	In-house workshop	20
16	Cheese head screw with hexagon socket, M3 ×12 mm	In-house workshop	21
16	Cheese head screw with hexagon socket, M3 × 20 mm	In-house workshop	22
16	Countersunk screws PH, M2 × 6 mm	In-house workshop	23

Quant.	Part	Purchased from	No.
1	D-SUB plug, 25-pin	Reichelt elektronik GmbH & Co. KG	
1	Aluminium plate 500×300 mm×3mm	In-house workshop	
	Several different M3 screws and nuts for the fixation on the aluminium plate	In-house workshop	
	**For the Arduino case:**		
1	Arduino case	3D-printed	24
1	Lower lid	3D-printed	25
1	Upper lid	3D-printed	26
1	Keypad cover	3D-printed	27
1	HIMALAYA basic MEGA 2560	Eckstein GmbH	28
1	Ramps 1.4 controller	Eckstein GmbH	
1	Character 20x4 LCD display module	Eckstein GmbH	29
1	4 × 4 matrix array keypad 8 pin 16 key membrane keyboard for Arduino	Eckstein GmbH	30
1	D-SUB socket, 25-pin	Reichelt elektronik GmbH & Co. KG	31
1	On-off switch, DC-socket	In-house workshop	32
1	DC-socket	In-house workshop	33
2	Machine screws PH, M2,5 × 5	In-house workshop	34
8	Machine screws PH, M3 × 10	In-house workshop	35
5	Machine screws PH, M3 × 8	In-house workshop	36
4	Machine screws PH, M3 × 6	In-house workshop	37
3	Machine screws PH, M3 × 25	In-house workshop	38
1	D-SUB extension cable, 1:1, 25-pin	Reichelt Elektronik GmbH & Co. KG	
1	12 V DC, 4 A power supply	In-house workshop	
4	DRV8825 Stepper Motor Driver	Eckstein GmbH	
1	4-Pin 20cm Jumper Wire Cable Female to Female	Eckstein GmbH	
1	Jumper Wire 10x1P female to female 20cm	Eckstein GmbH	

Wiring of the Arduino and pumps: For the connection of the Arduino with the pumps and the end stop switch we used a RAMPS 1.4 controller, which was attached to the Arduino Mega 2560. The DRV8825 Stepper Motor Driver was clipped to the RAPMS controller at *x*, *y*, *z* and E0 (take care of the right direction!) without using any jumpers. The assignment of the stepper motors, end stop switches, display and keypad are shown in Figure 3. The RAMPS controller was connected to a standard 12 V DC, 4 A power supply. Be careful, not to connect the power supply and the USB of the Arduino at the same time!

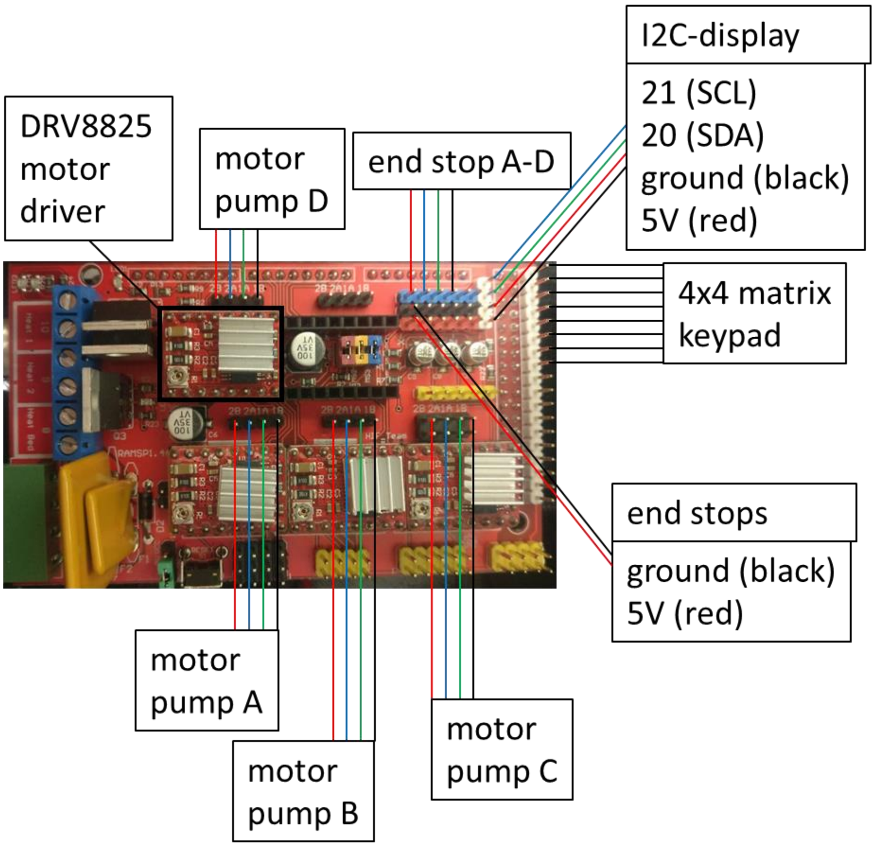

Figure S3: Wiring diagram of the syringe pump rack.

Figure S2d shows, how the Arduino and the RAMPS controller was mounted in the 3D-printed case. The connections from the controller were passed to a 25-pin D-Sub socket. Table S2 shows the pin assignment of the 25-pin socket to the RAMPS controller and the 25-pin plug to the pumps, which are connected through a D-SUB extension cable 1:1.

Table S2: Pin assignment of the 25-pin D-Sub connections.

Pin	Pin socket on RAMPS	Pin plug connected to
1	2B, X Driver	Motor A, red
2	2A, X Driver	Motor A, black
3	1A, X Driver	Motor A, green
4	1B, X Driver	Motor A, black
5	2B, Y Driver	Motor B, red
6	2A, Y Driver	Motor B, black
7	1A, Y Driver	Motor B, green
8	1B, Y Driver	Motor B, black
9	2B, Z Driver	Motor C, red
10	2A, Z Driver	Motor C, black
11	1A, Z Driver	Motor C, green
12	1B, Z Driver	Motor C, black
14	2B, E0 Driver	Motor D, red
15	2A, E0 Driver	Motor D, black
16	1A, E0 Driver	Motor D, green
17	1B, E0 Driver	Motor D, black
18	5V	Stop switches VCC
20	GND	Stop switches GND
22	X-	Stop switch A
23	X+	Stop switch B
24	Y-	Stop switch C
25	Y+	Stop switch D

More information and datasheets for the Arduino and RAMPS controller can be found at https://reprap.org/wiki.

Programming with Arduino software: The software was written on the open-source Arduino software. If the motors and end stops are connected as mentioned above, the written software can be used instantly, otherwise the pins of the Arduino have to be edited to the corresponding pins. The syringes, for which the software was written, are shown in Table S3. First, the approximate mm/mL was measured for each syringe and put in line 40 (size of the syringes) and 41 (mm/mL of each syringe) of the code:

line 40: *int syringe[6] = {1, 2, 5, 10, 20, 50};*

line 41: *float syringefactor[6] = {57.3, 16.4, 8.38, 4.97, 3.12, 1.494};*

After the first accuracy test (see below) the syringefactors was corrected corresponding to the measured volumes:

line 41: *float syringefactor[6] = {58.310, 16.616, 8.459, 5.130, 3.200, 1.548};*

With this method different syringes can be insert in the code and a very accurate dispensing is achievable. The complete program code can be found in the zip-file (Supporting Information File 2).

Brief instruction for using the syringe pump rack: The keys A–D start and stop the pumps A–D. With the keys 1–4 you get to the syringe menu, where the size of the syringes can be change by pressing 1 or 7 (up and down). With the #-key you go to the flow rate entry, where flow rates can be set in ranges from 1–9999 µL/min. By pressing the *-key all entries in the syringe menu are saved and you return to the main menu. During change of the size and flow rate all pumps stop!

The display will not refresh automatically; you have to press the 0-key to refresh the display manually. After refresh, it shows the actual dispensed volume of each syringe. With the keys 5–8 you can reset the dispensed volume to 0 of the pumps A–D.

Accuracy test of the syringe pump: Each kind of syringe was tested with typical flow rates for continuous flow reactions. Distilled water was dispensed at room temperature for 4 min, weight and compared to the theoretical volume. In Table S3 you see the measured weight. With all syringes we never exceed +- 1% of deviation, which is comparable to commercial available syringe pumps.

Table S3: Accuracy test of the used syringes.

**Volume**	**Syringe**	**Flow rate [µL/min]**	**Weigth [mg]**	**Deviation**	**End-stop position**
1 mL	HSW NORM-JECT®	50	200	0%	1 dot
2 mL	BD Discardit II	100	401	+ 0.25%	2 dots
5 mL	BD Discardit II	150	601	+ 0.17%	3 dots
10 mL	HSW NORM-JECT®	200	799	– 0.125%	3 dots
20 mL	BD Discardit II	500	2014	+ 0.7%	3 dots
50 mL	HSW NORM-JECT®	1000	4005	+ 0.125%	4 dots

General information

For flow reactions the self-built syringe pump rack was used and, if necessary, additional syringe pumps LA-30 from Landgraf Laborsysteme HLL GmbH were used. Dry CH_2Cl_2 and DMF were distilled from P_4O_{10}. Dry solvents were stored over molecular sieves under an atmosphere of nitrogen. For reaction monitoring TLC plates from Macherey-Nagel “Polygram Sil G/UV254” were used. For the mixing test solutitons of Sudan Red G and Iron(II)phthalocyanine in DCM/Pyridine 99/1 were used. Preparative column chromatography was performed using a LaFlash system (VWR) and a flow rate of 20 mL/min with EasyVarioFlash cartridges from Götec-Labortechnik. Silica 60 (0.04–0.063 mm) from Macherey-Nagel was used, solvents used as eluents were of technical grade and distilled prior to their use. Petroleum ether (PE) refers to the fraction boiling at 60–90 °C. NMR spectra were recorded on a Bruker “Avance 400” spectrometer and calibrated to the residual solvent signal ($CDCl_3$: ^{1}H 7.27 ppm, ^{13}C 77.0 ppm). For peak assignment additional NMR spectra (DEPT-135, ^{1}H,^{1}H-COSY, ^{1}H,^{13}C-HMBC, ^{1}H,^{13}C-HSQC) were used, atoms are numbered according to the carbohydrate nomenclature.

Experimental

Flow synthesis of 2,3,4,6-tetra-*O*-acetyl-α-D-glucopyranosyl bromide (2)

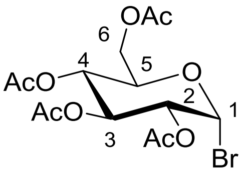

A solution of β-D-glucose pentaacetate (**1**, 1 M in dry CH_2Cl_2, 1 equiv, 107 μL/min) and a solution of HBr (33% in glacial acetic acid, 5 equiv, 93 μL/min) were mixed and pumped through flow reactor R3 with a total reactor volume of 1.5 mL at rt, resulting in a total flow rate of 200 μL/min and a residence time of 7.5 min. The reaction was quenched by pumping water through a third inlet with a flow rate of 200 μL/min. The biphasic mixture was passed through a CSTR (V = 1 mL) with an in-printed stirring bar. The phases were separated under flow conditions in a 10 mL syringe and the organic phase was passed through a second syringe containing a $NaHCO_3$-solution for neutralization. Through an outlet in the cap, the excess water and formed CO_2 was removed. The organic phase was collected, dried over Na_2SO_4, filtered and concentrated in vacuo. The yellowish residue was washed with a mixture of PE and EtOAc (1:1) to afford glucopyranosyl bromide **2** as a white solid with a yield of 86% based on the steady state. R_f = 0.71 (PE:EtOAc, 1:1).

^{1}H NMR (400 MHz, $CDCl_3$): δ 6.61 (d, $J_{1,2}$ = 4.0 Hz, 1H, 1-H), 5.57 (app. t, J = 9.7 Hz, 1H, 3-H), 5.17 (app. t, J = 10.3 Hz, 1H, 4-H), 4.85 (dd, $J_{2,3}$ = 10.0 Hz, $J_{1,2}$ = 4.0 Hz, 1H, 2-H) 4.36 – 4.28 (m, 2H, 5-H, 6-H_a), 4.13 (d, $J_{6b,6a}$ = 10.0 Hz, 6-H_b), 2.11, 2.10, 2.06, 2.04 (4 s, 12 H, 4 × $COCH_3$) ppm.

^{13}C NMR (100 MHz, $CDCl_3$): δ 170.5, 169.8, 169.8, 169.4 (4 × $COCH_3$), 86.5 (C1), 72.1 (C5), 70.6 (C2), 70.1 (C3), 67.1 (C4), 60.9 (C6), 20.6, 20.6, 20.6, 20.5 (4 × $COCH_3$) ppm. Spectroscopic data are in agreement with reported literature.[1]

Flow synthesis of methyl 2,3,4,6-tetra-*O*-acetyl-β-D-glucopyranoside (3) via Koenigs-Knorr glycosylation

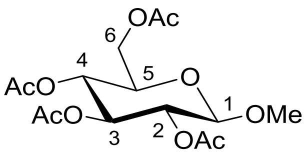

A solution of 2,3,4,6-tetra-*O*-acetyl-α-D-glucopyranosyl bromide (**2**, 0.25 M in dry CH_2Cl_2, 1 equiv, 4.67 g, 166 μL/min) and MeOH (HPLC grade, 20 equiv, 34 μL/min) were mixed and pumped through a CSTR with a total reactor volume of 1.0 mL followed by a packed-bed column equipped with a mixture of AgOTf (5.83 g, 2 equiv) and molecular sieves (4 Å). The total flow rate was 200 μL/min and the residence time was 5 min. The reaction mixture was collected in a glass vial containing sat. $NaHCO_3$ solution. The phases were separated and the organic phase was dried over Na_2SO_4, filtered and concentrated in vacuo. Flash column chromatography (PE/EtOAc, 4:1) afforded methyl glycoside **3** (1.81 g) as yellowish oil with a yield of 44%. R_f = 0.49 (PE:EtOAc 1:1).

^{1}H NMR (400 MHz, $CDCl_3$): δ 5.20 (app. t, J = 9.5 Hz, 1H, 3-H), 5.09 (app. t, J = 9.7 Hz, 1H, 4-H), 4.98 (dd, $J_{2,1}$ = 7.9 Hz, $J_{2,3}$ = 9.5 Hz, 1H, 2-H), 4.43 (d, $J_{1,2}$ = 7.9 Hz, 1H, 1-H), 4.27 (dd, $J_{6a,6b}$ = 12.3 Hz, $J_{6a,5}$ = 4.6 Hz, 1H, 6-H_a), 4.14 (dd, $J_{6b,6a}$ = 12.3 Hz, $J_{6b,5}$ = 2.3 Hz, 1H, 6-H_b), 3.70 (ddd, $J_{5,4}$ = 9.9 Hz, $J_{5,6a}$ = 4.5 Hz, $J_{5,6b}$ = 2.3 Hz, 1H, 5-H), 3.50 (s, 3H, OCH_3), 2.08, 2.04, 2.02, 1.99 (4 s, 12H, 4 × COCH_3) ppm.

^{13}C NMR (100 MHz, $CDCl_3$): δ 170.6, 170.2, 169.4, 169.4 (4× O*C*OCH$_3$), 101.6 (C-1), 72.8 (C-2), 71.7 (C-3), 71.2 (C-4), 68.4 (C-5), 61.9 (C-6), 57.0 (O*C*H$_3$), 20.7, 20.6, 20.5, 20.5 (4 × CO*C*H$_3$) ppm.

Spectroscopic data are in agreement with reported literature.[2]

Batch synthesis of 2,3,4,6-tetra-*O*-acetyl-D-glucopyranose (4)

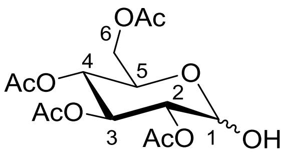

A solution of β-D-glucose pentaacetate (**1**, 1.95 g, 5 mmol) and hydrazine acetate (0.55 g, 6 mmol) in dry DMF (5 mL) was stirred for 45 min at 50 °C. Afterwards, the reaction mixture was diluted with ethyl acetate (5 mL) and washed with water (3 × 10 mL) and brine (10 mL). The organic phase was dried over Na_2SO_4 and concentrated in vacuo. Coevaporation with

n-heptane afforded title compound **4** (1.69 g, 97%, 72:28 α:β anomeric ratio) as a colourless foam. R_f = 0.54 (PE:EtOAc, 1:1).

^{1}H NMR (400 MHz, $CDCl_3$): δ 5.53 (app. t, 1H, J = 9.9 Hz, 3-H_α), 5.44 (d, 1H, $J_{1,2}$ = 3.5 Hz, 1-H_α), 5.23 (app. t, 1H, J = 9.6 Hz, 3-H_β), 5.11–5.05 (m, 2H, J = 9.6 Hz, 4-$H_{\alpha/\beta}$), 4.90–4.85 (m, 2H, 2-$H_{\alpha/\beta}$), 4.73 (d, 1H, $J_{1,2}$ = 8.1 Hz, 1-H_β), 4.29-4.21 (m, 3H, 5-H_α, 6a-$H_{\alpha/\beta}$), 4.17-4.11 (m, 2H, 6b-$H_{\alpha/\beta}$), 3.76 (ddd, 1H, $J_{5,4}$ = 10.1 Hz, $J_{5,6a}$ = 4.8 Hz, $J_{5,6b}$ = 2.5 Hz, 5-H_β), 2.09, 2.08, 2.08, 2.03, 2.03, 2.01 (12H, COCH_3) ppm.

^{13}C NMR (100 MHz, $CDCl_3$): δ 170.8, 170.8, 170.2, 170.1, 169.6, 169.53, (*CO*-CH_3), 95.5 (*C*-1_β), 90.1 (*C*-1_α), 73.2 (*C*-3_β), 72.2 (*C*-5_β), 72.0 (*C*-2_β), 71.1 (*C*-2_α), 69.9 (*C*-3_α), 68.5, 68.4 (*C*-$4_{\alpha/\beta}$), 67.1 (*C*-5_α), 61.9, (*C*-$6_{\alpha/\beta}$), 20.7, 20.7, 20.7, 20.6, 20.5, (COCH_3) ppm.

Spectroscopic data are in agreement with reported literature.[3]

Flow synthesis of 2,3,4,6-tetra-*O*-acetyl-α-D-glucopyranosyl trichloroacetimidate (5)

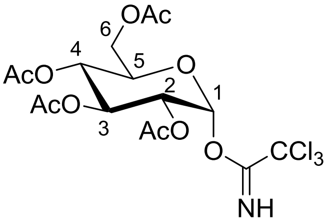

A solution of 2,3,4,6-tetra-*O*-acetyl-β-D-glucopyranose (**4**, 0.1 M in dry CH_2Cl_2, 1 equiv, μL/min), a solution of trichloroacetonitrile (1 M in dry CH_2Cl_2, 10 eq, μL/min) and a solution of DBU (0.1 M in dry CH_2Cl_2, 0.3 eq, μL/min) were mixed and pumped through flow reactor R1 with a total reactor volume of 1.05 mL at 0 °C, resulting in a total flow rate of 300 μL/min and a residence time of 3.5 min. The reaction mixture was collected in a glass vial containing sat. $NaHCO_3$ solution. The phases were separated and the organic phase was dried over Na_2SO_4, filtered and concentrated in vacuo. Flash column chromatography (PE/EtOAc, 9:1 + 2% NEt_3) afforded glucopyranosyl imidate **5** as a yellowish oil in a yield of 67% based on the steady state. R_f = 0.65 (PE/EtOAc, 1:1).

^{1}H NMR (400 MHz, $CDCl_3$): δ 8.70 (s, 1H, N*H*), 6.56 (d, $J_{1,2}$ = 3.7 Hz, 1H, 1-H), 5.57 (app. t, $J_{2,3}$ = 9.9 Hz, 1H, 3-H), 5.18 (app. t, J = 9.6 Hz, 1H, 4-H), 5.13 (dd, $J_{2,3}$ =10.1 Hz, $J_{2,1}$ = 3.6 Hz, 1H, 2-H) 4.28 (dd, $J_{6a,6b}$ = 12.2 Hz, $J_{6a,5}$ = 4.0 Hz, 1H, 6-H_a), 4.22 (ddd, $J_{5,4}$ =10.2 Hz, $J_{5,6a}$ = 4.1 Hz, $J_{5,6b}$ = 1.9 Hz, 1H, 5-H), 4.13 (dd, $J_{6b,6a}$ = 12.3 Hz, $J_{6b,5}$ = 2.0 Hz, 1H, 6-H_b), 2.08, 2.05, 2.04, 2.02 (4 s, 12H, 4 × COCH_3) ppm.

^{13}C NMR (100 MHz, $CDCl_3$): δ 170.5, 170.0, 169.8, 169.5 (4 × *C*OCH_3), 160.8 (*C*NH-CCl_3), 92.9 (C-1), 90.7 (*C*Cl_3), 70.0 (C-5), 69.8 (C-3), 69.7 (C-2), 67.8 (C-4), 61.3 (C-6), 20.6, 20.6, 20.6, 20.4 (4 × CO*C*H_3) ppm.
Spectroscopic data are in agreement with reported literature.[4]

Multistep synthesis of methyl 2,3,4,6-tetra-*O*-acetyl-β-D-glucopyranoside (3)

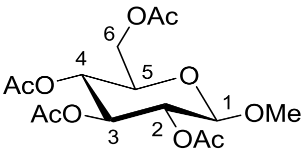

A solution of 2,3,4,6-tetra-*O*-acetyl-D-glucopyranose (**4**, 0.1 M in dry CH_2Cl_2, 1 equiv, 67 μL/min), a solution of trichloroacetonitrile (1 M in dry CH_2Cl_2, 10 equiv, 67 μL/min) and a solution of DBU (0.1 M in dry CH_2Cl_2, 0.2 equiv, 13 μL/min) were mixed and pumped at rt through the first flow reactor R1 with a total reactor volume of 1.05 mL. The resulting reaction mixture was pumped at 0 °C together with a solution of TMSOTf (0.01 M in CH_2Cl_2, 0.3 equiv, 20 μL/min) and a solution of methanol (0.1 M in CH_2Cl_2, 20 equiv, 133 μL/min) through the second flow reactor R1 resulting in a total flow rate of 300 μL/min and a residence time of 3.5 min. The reaction mixture was collected in a glass vial containing a sat. $NaHCO_3$ solution. The phases were separated and the organic phase was dried over Na_2SO_4, filtered and concentrated in vacuo. Flash column chromatography (PE/EtOAc 3:1) afforded methyl glycoside **3** as a yellowish oil in a yield of 58% based on the steady state R_f = 0.46 (PE/EtOAc 1:1).
The NMR data are shown above.

Multistep synthesis of propargyl 2,3,4,6-tetra-*O*-acetyl-β-D-glucopyranoside (6)

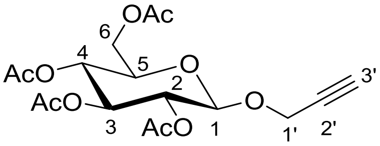

A solution of 2,3,4,6-tetra-*O*-acetyl-β-D-glucopyranoside (**4**, 0.1 M in dry CH_2Cl_2, 1 equiv, 56 μL/min), a solution of trichloroacetonitrile (1 M in dry CH_2Cl_2, 10 equiv, 56 μL/min) and a solution of DBU (0.1 M in dry CH_2Cl_2, 0.5 equiv, 28 μL/min) were mixed and pumped at rt through the first flow reactor R1 with a total reactor volume of 1.05 mL. The resulting reaction mixture was pumped at 0 °C together with a solution of TMSOTf (0.1 M in CH_2Cl_2, 0.7 equiv, 56 μL/min) and a solution of propargyl alcohol (1 M in CH_2Cl_2, 10 equiv, 56

μL/min) through the second flow reactor R1 resulting in a total flow rate of 252 μL/min and a residence time of 4.2 min. The reaction mixture was collected in a glass vial containing a sat. $NaHCO_3$ solution. The phases were separated and the organic phase was dried over Na_2SO_4, filtered and concentrated in vacuo. Flash column chromatography (PE/EtOAc, 4:1) afforded propargyl glycoside **6** as a yellowish oil in a yield of 43% based on the steady state. R_f = 0.59 (PE/EtOAc, 1:1).

^{1}H NMR (400 MHz, $CDCl_3$): δ 5.25 (app. t, J = 9.4 Hz, 1H, 2-H), 5.11 (app. t, J = 9.7 Hz, 1H, 3-H), 5.02 (dd, $J_{4,5}$ = 9.5 Hz, J = 7.9 Hz, 1H, 4-H), 4.79 (d, $J_{1,2}$ = 7.9 Hz, 1H, 1-H), 4.39 (s, 1H, 1‘-H_a), 4.38 (s, 1H, 1‘-H_b), 4.28 (dd, $J_{6a,6b}$ = 12.3 Hz, $J_{6a,5}$ = 4.6 Hz, 1-H, 6-H_a), 4.16 (dd, $J_{6a,6b}$ = 12.3 Hz, $J_{6b,5}$ = 2.5 Hz, 6-H_b), 3.74 (ddd, $J_{5,4}$ = 10.3 Hz, $J_{5,6a}$ = 4.6 Hz, $J_{5,6b}$ = 2.4 Hz, 1H, 5-H), 2.48 (app. t, J = 2.4 Hz, 1H, 3‘-H), 2.10, 2.07, 2.04, 2.02 (4 s, 12H, 4 × COCH_3) ppm.

^{13}C NMR (100 MHz, $CDCl_3$): δ 170.7, 170.3, 169.4, 169.4 (4 × *C*OCH3), 98.1 (C-1), 78.1 (C-2‘), 75.5 (C-3‘), 72.7 (C-2), 71.9 (C-3), 70.9 (C-4), 68.3 (C-5), 61.7 (C-6), 55.9 (C-1‘), 20.7, 20.7, 20.6, 20.6 (4 × CO*C*H_3) ppm.

Spectroscopic data are in agreement with reported literature.[5]

Multistep synthesis of 4-pentynyl 2,3,4,6-tetra-*O*-acetyl-β-D-glucopyranoside (7)

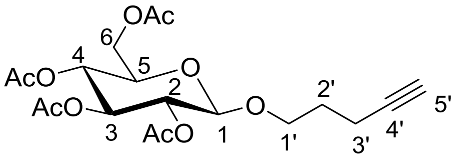

The same procedure as for the preparation of propargyl glycoside **6** was used for the synthesis of 4-pentynyl glucopyranoside **7** with 4-pentynol as alcohol. The product was obtained as a yellowish oil in a yield of 69% based on the steady state. R_f = 0.35 (PE/EtOAc, 1:1).

^{1}H-NMR (400 MHz, $CDCl_3$): δ 5.17 (app. t, J = 9.5 Hz, 1H, 3-H), 5.04 (app. t, J = 9.7 Hz, 1H, 4-H), 4.94 (dd, $J_{2,3}$ = 9.7 Hz, $J_{2,1}$= 7.9 Hz, 1H, 2-H), 4.47 (d, $J_{1,2}$ = 7.9 Hz, 1H, 1-H), 4.23 (dd, $J_{6a,6b}$ = 12.2 Hz, $J_{6a,5}$ = 4.6 Hz, 1H, 6-H_a), 4.09 (dd, $J_{6b,6a}$ = 12.2 Hz, $J_{6b,5}$ = 2.4 Hz, 1H, 6-H_b), 3.92 (dt, $J_{1'a,1'b}$ = 9.8 Hz, $J_{1'a,2'}$ = 5.4 Hz, 1H, 1‘-H_a), 3.67 (ddd, $J_{5,4}$ = 9.9 Hz, $J_{5,6a}$ = 4.6 Hz, $J_{5,6b}$ = 2.4 Hz, 1H, 5-H), 3.59 (ddd, $J_{1'b,1'a}$ = 9.7 Hz, J = 8.2 Hz, J = 5.1 Hz, 1H, 1‘-H_b), 2.23–2.19 (m, 2H, 3‘-H), 2.04, 2.01, 1.98, 1.96 (4 s, 12H, 4 × COCH_3), 1.91 (t, $J_{5',6'}$ = 2.6 Hz, 1H, 5‘-H), 1.84–1.68 (m, 2H, 2‘-H) ppm.

^{13}C-NMR (100 MHz, $CDCl_3$): δ 170.5, 170.1, 169.3, 169.2 (4 × *C*OCH_3), 100.9 (C-1), 83.2 (C-4‘), 72.6 (C-3), 71.6 (C-2), 71.2 (C-4), 68.7 (C-5‘), 68.3 (C-1‘), 68.2 (C-5), 61.8 (C-6), 28.1 (C-2‘), 20.6, 20.5, 20.5, 20.5, (4 × CO*C*H_3), 14.6 (C-3‘) ppm.
Spectroscopic data are in agreement with reported literature.[6]

Flow synthesis of 2,3,4,6-tetra-*O*-acetyl-β-D-glucopyranosyl azide (8)

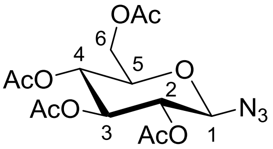

A solution of β-D-glucose pentaacetate (**1**, 0.5 M in dry CH_2Cl_2, 1 equiv, 27 μL/min), a solution of trimethylsilyl azide (1 M in CH_2Cl_2, 4 equiv, 55 μL/min) and a solution of $SnCl_4$ (0.2 M in CH_2Cl_2, 1 equiv, 68 μL/min) were mixed and pumped through flow reactor R1 with a total reactor volume of 1.05 mL at rt, resulting in a total flow rate of 150 μL/min and a residence time of 7 min. The reaction mixture was collected in a glass vial containing sat. $NaHCO_3$ solution. The phases were separated and the organic phase was dried over Na_2SO_4, filtered and concentrated in vacuo. Flash column chromatography (toluene/EtOAc, 9:1) afforded glycosyl azide **8** as a white solid in a yield of 80% based on the steady state. R_f = 0.55 (PE/EtOAc, 1:1).
^{1}H NMR (400 MHz, $CDCl_3$): δ 5.23 (app.t, $J_{3,2}$ =9.5 Hz, $J_{3,4}$ = 9.4 Hz, 1H, 3-H), 5.12 (app. t, $J_{4,5}$ = 9.9 Hz, $J_{4,3}$ = 9.5 Hz, 1H, 4-H), 4.97 (app.t, $J_{2,3}$ = 9.5 Hz $J_{2,1}$ = 8.9 Hz, 1H, 2-H), 4.66 (d, $J_{1,2}$ = 8.8 Hz, 1H, 1-H), 4.29 (dd, $J_{6a,6b}$ =12.4 Hz, $J_{6a,5}$ = 4.7 Hz, 1H, 6-H_a), 4.19 (dd, $J_{6b,6a}$ = 12.4 Hz, $J_{6b,5}$ = 2.2 Hz, 1H, 6-H_b), 3.80 (ddd, $J_{5,4}$ = 10.0 Hz, $J_{5,6a}$ = 4.7 Hz, $J_{5,6b}$ = 2.3 Hz, 1H, 5- H), 2.12, 2.09, 2.04, 2.02 (4 s, 12H, 4 × COC*H*$_3$) ppm.
^{13}C NMR (100 MHz, $CDCl_3$): δ 170.6, 170.1, 169.3, 169.2 (4 × *C*OCH_3), 87.9 (C-1), 74.0 (C-5), 72.6 (C-3), 70.6 (C-2), 67.9 (C-4), 61.6 (C-6), 20.7, 20.6, 20.5 20.5 (4 × CO*C*H_3) ppm.
Spectroscopic data are in agreement with reported literature.[7]

NMR spectra

^{1}H NMR spectrum of 2,3,4,6-tetra-*O*-acetyl-α-D-glucopyranosyl bromide (2)

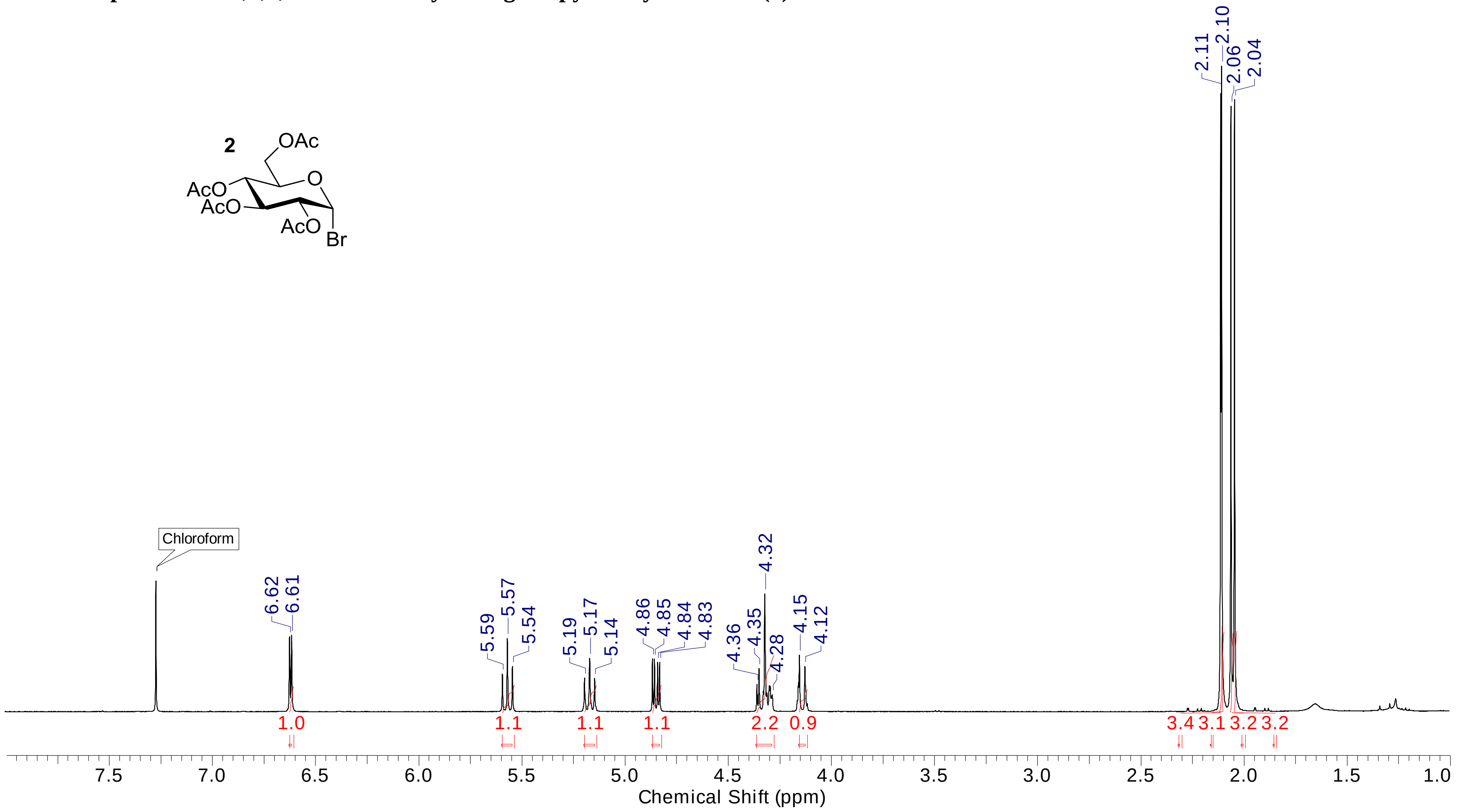

^{13}C NMR spectrum of 2,3,4,6-tetra-*O*-acetyl-α-D-glucopyranosyl bromide (2)

2

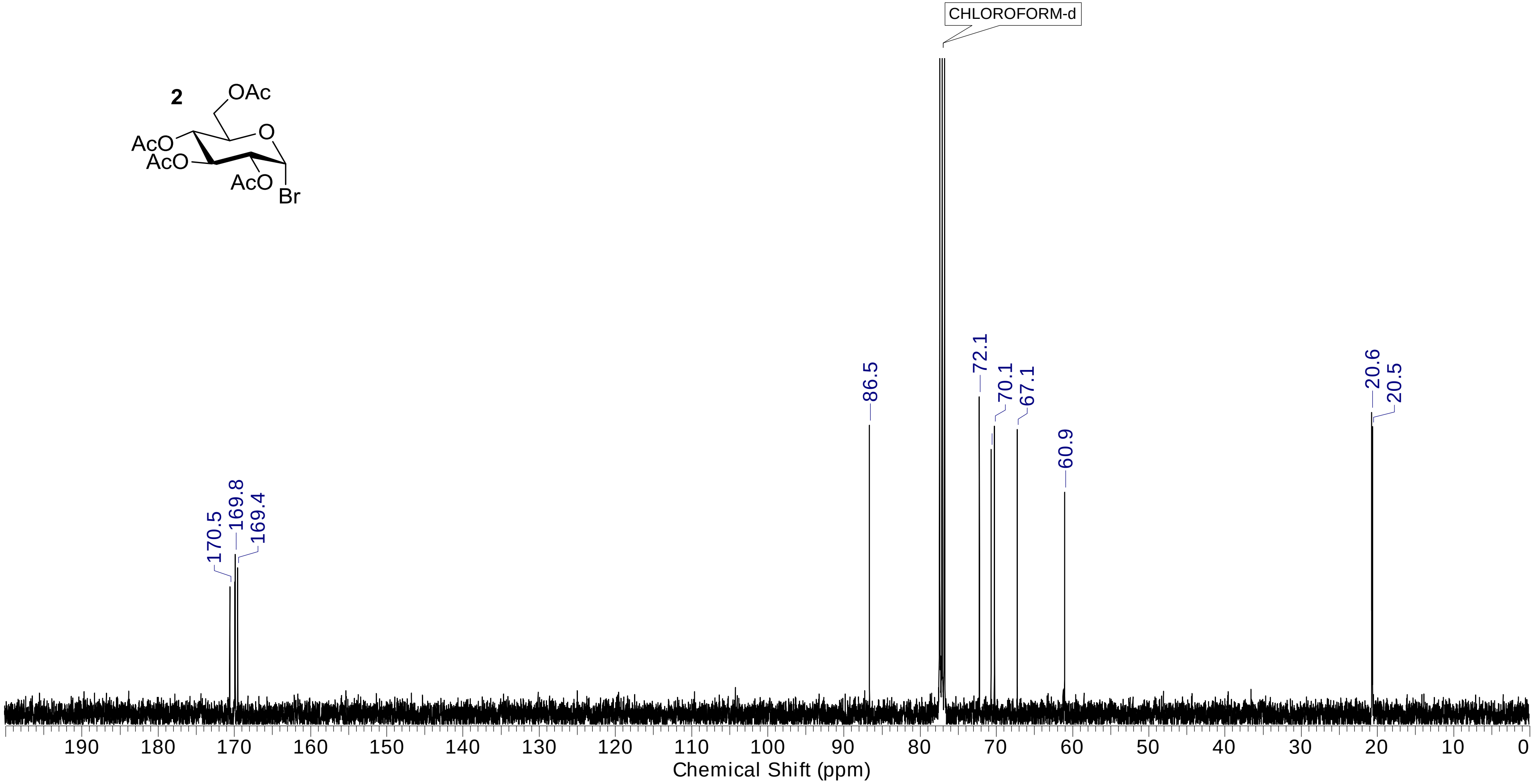

^{1}H NMR spectrum of methyl 2,3,4,6-tetraacetyl-β-D-glucopyranoside (3)

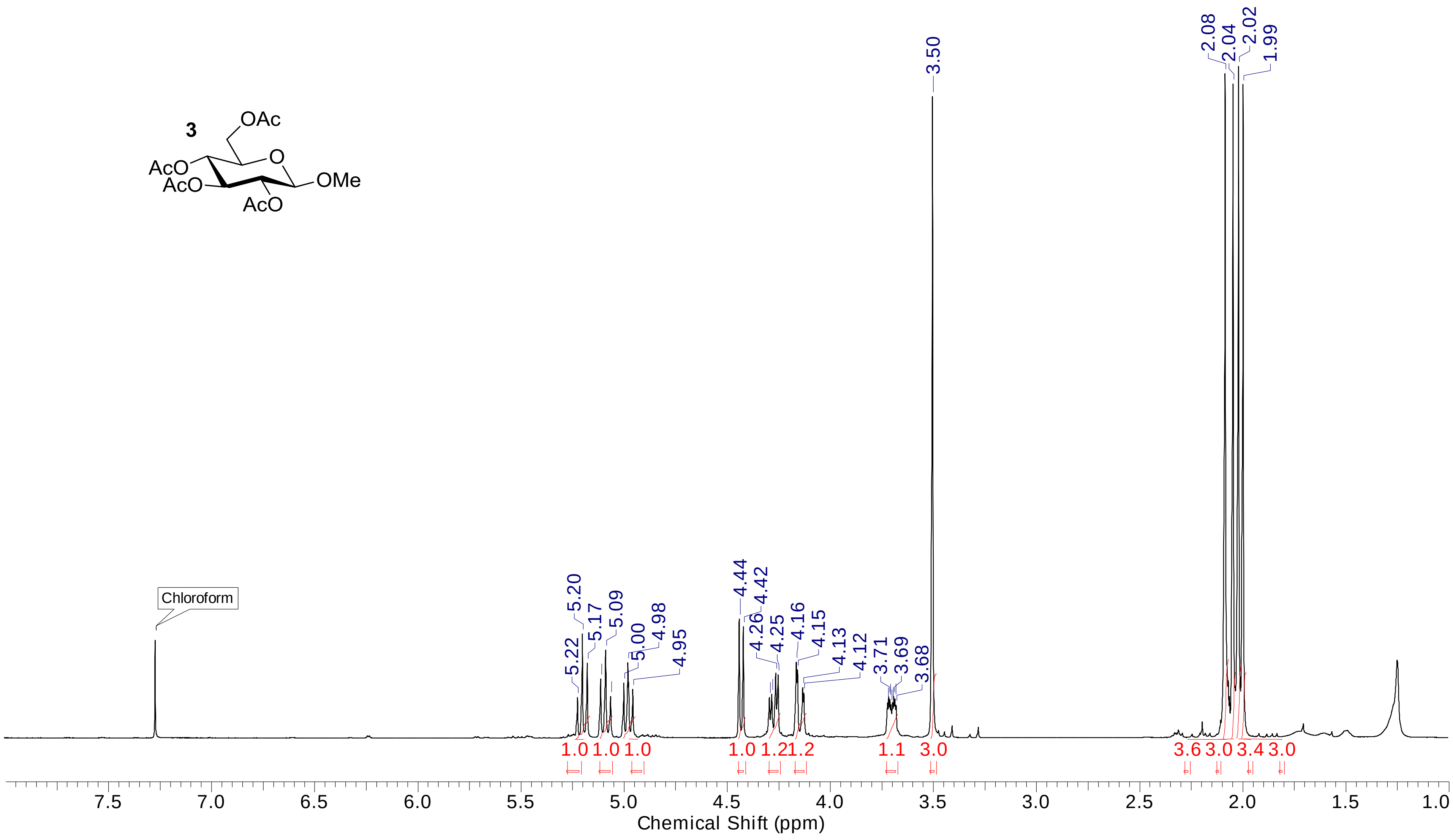

^{13}C NMR spectrum of methyl 2,3,4,6-tetraacetyl-β-D-glucopyranoside (3)

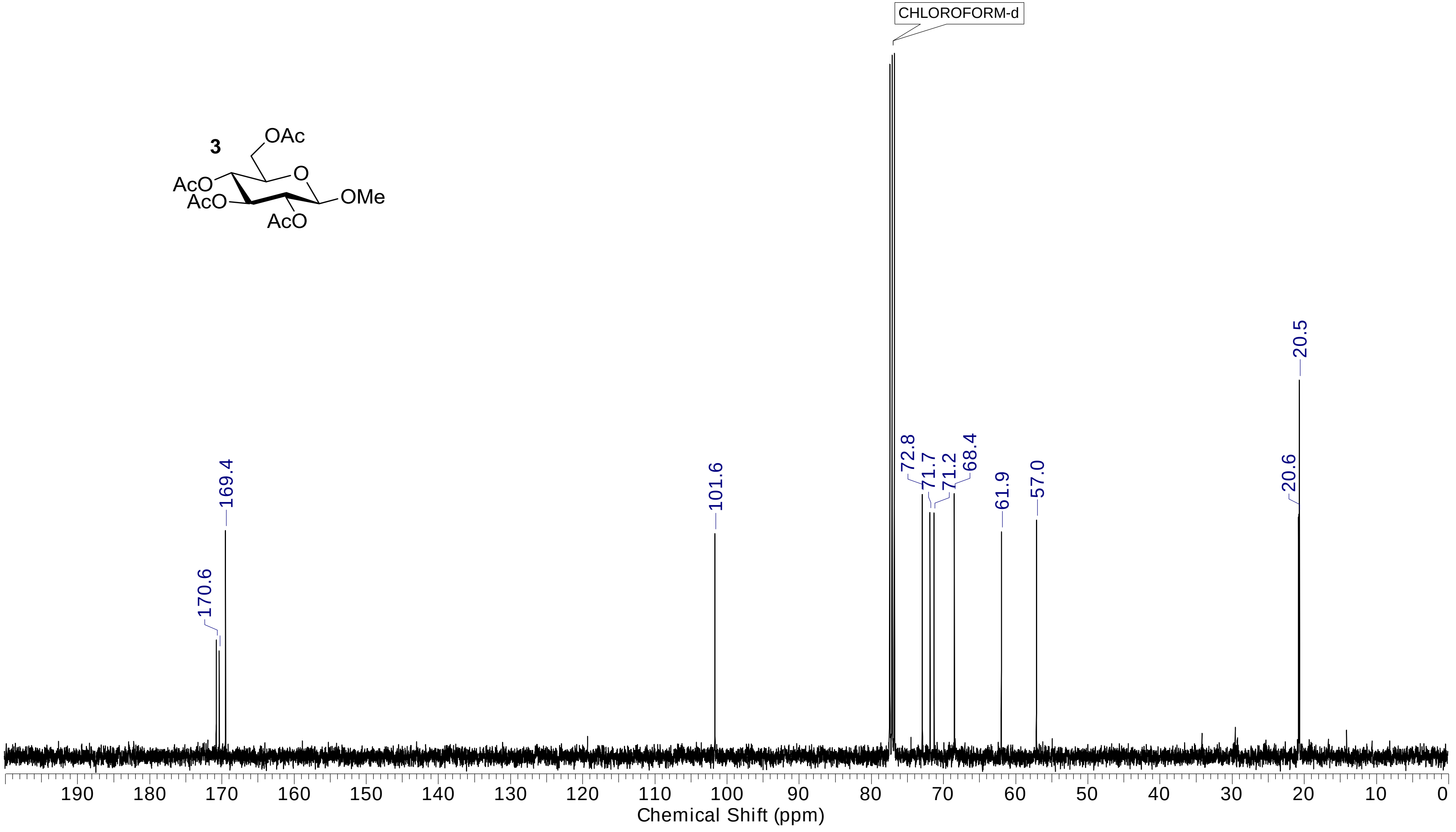

^{1}H NMR spectrum of 2,3,4,6-tetra-*O*-acetyl-D-glucopyranose (4)

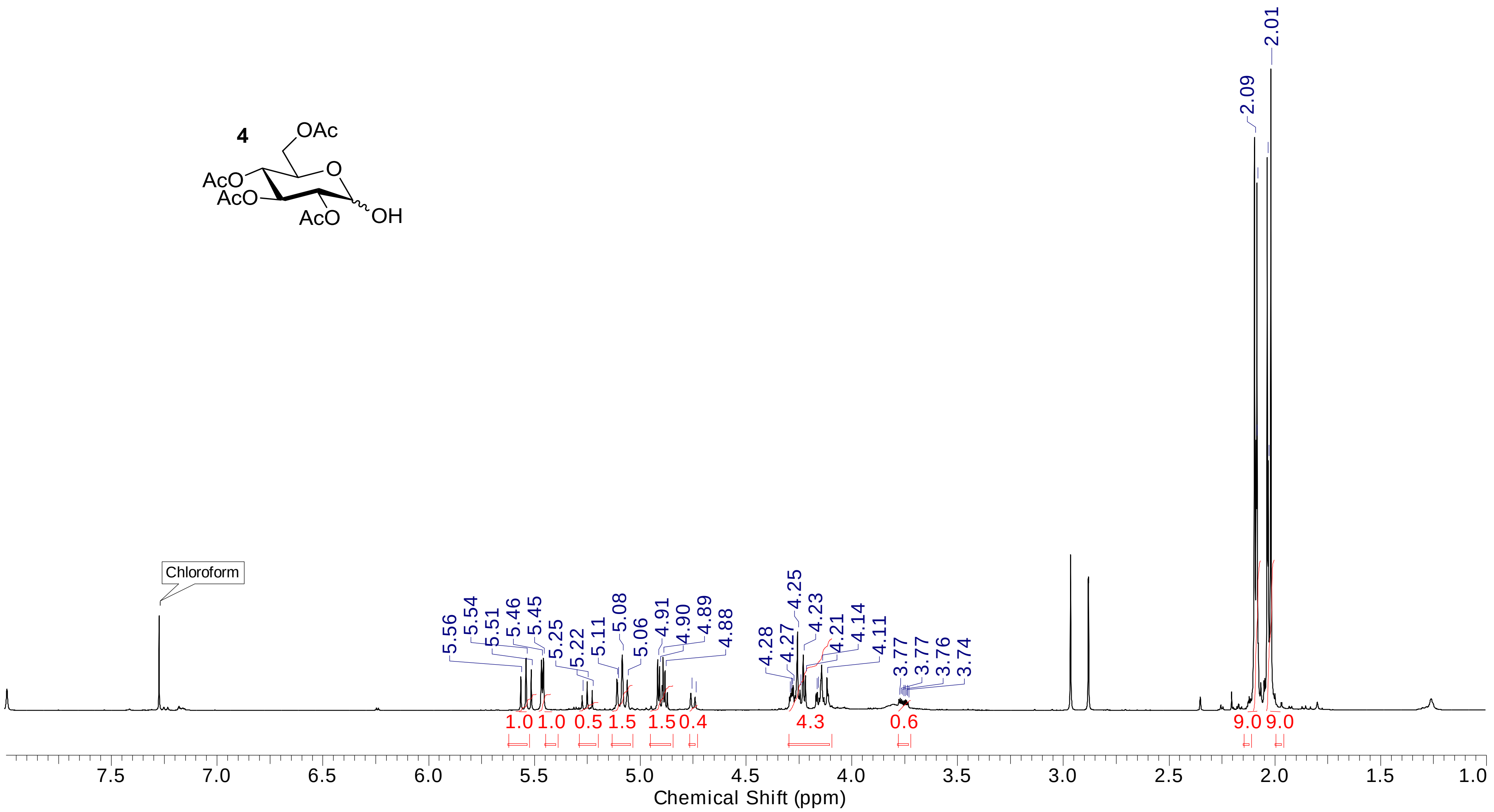

^{13}C NMR spectrum of 2,3,4,6-tetra-*O*-acetyl-D-glucopyranose (4)

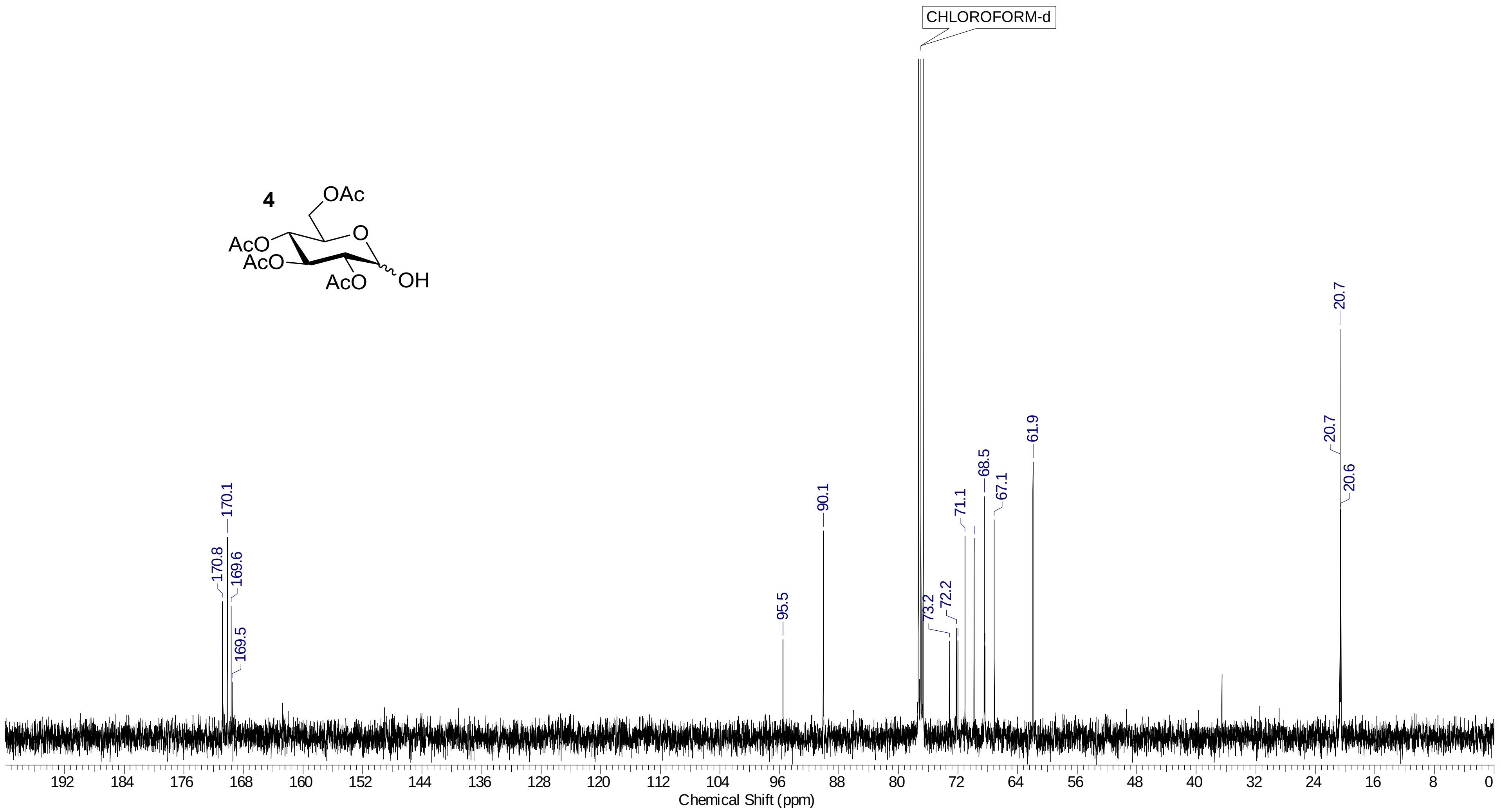

^{1}H NMR spectrum of 2,3,4,6-tetra-*O*-acetyl-α-D-glucopyranosyl trichloroacetimidate (5)

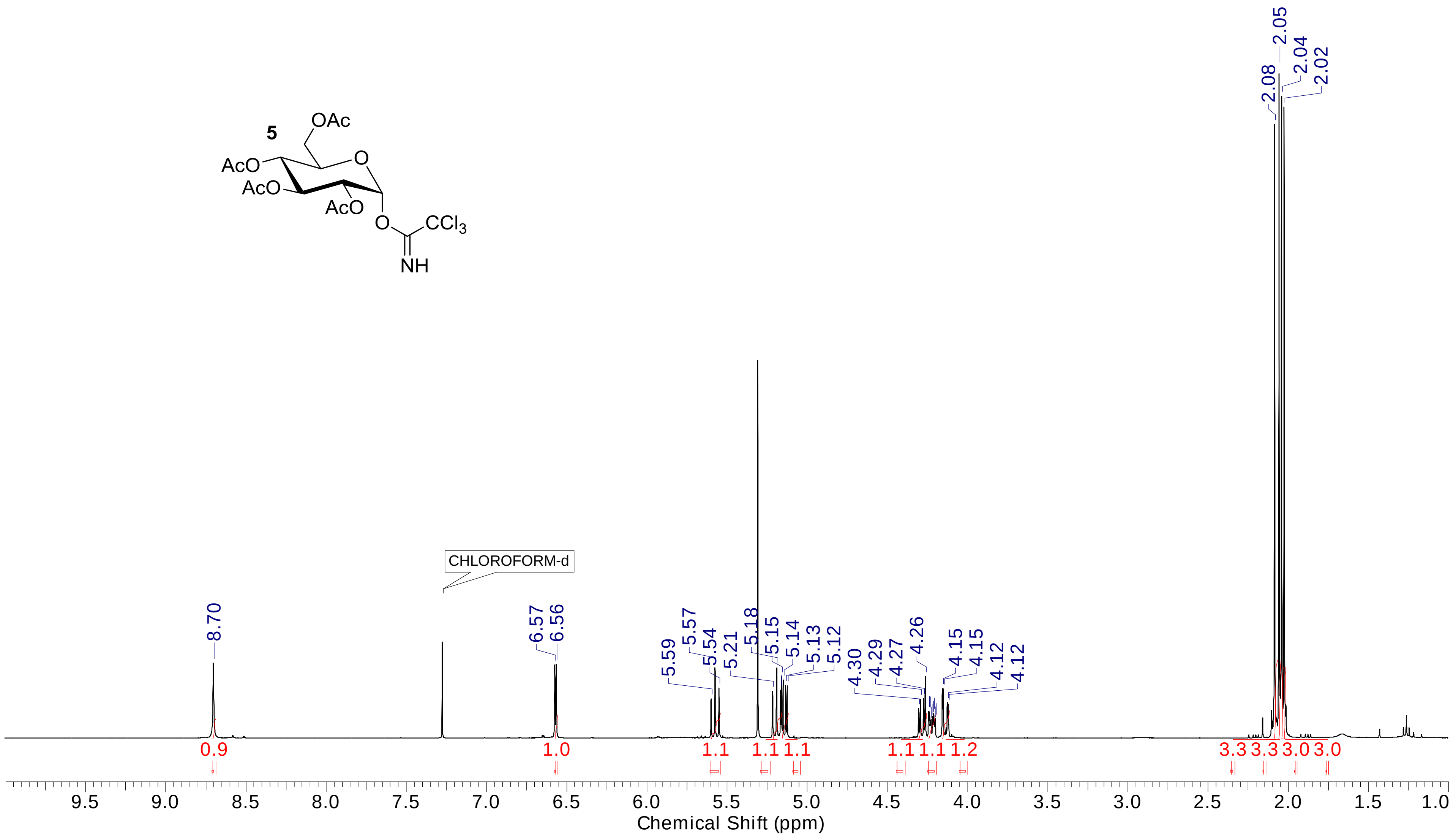

^{13}**C NMR spectrum of 2,3,4,6-tetra-*O*-acetyl-α-D-glucopyranosyl trichloroacetimidate (5)**

5

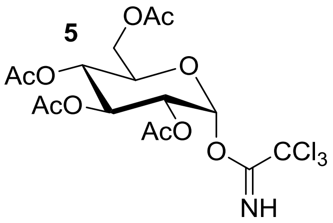

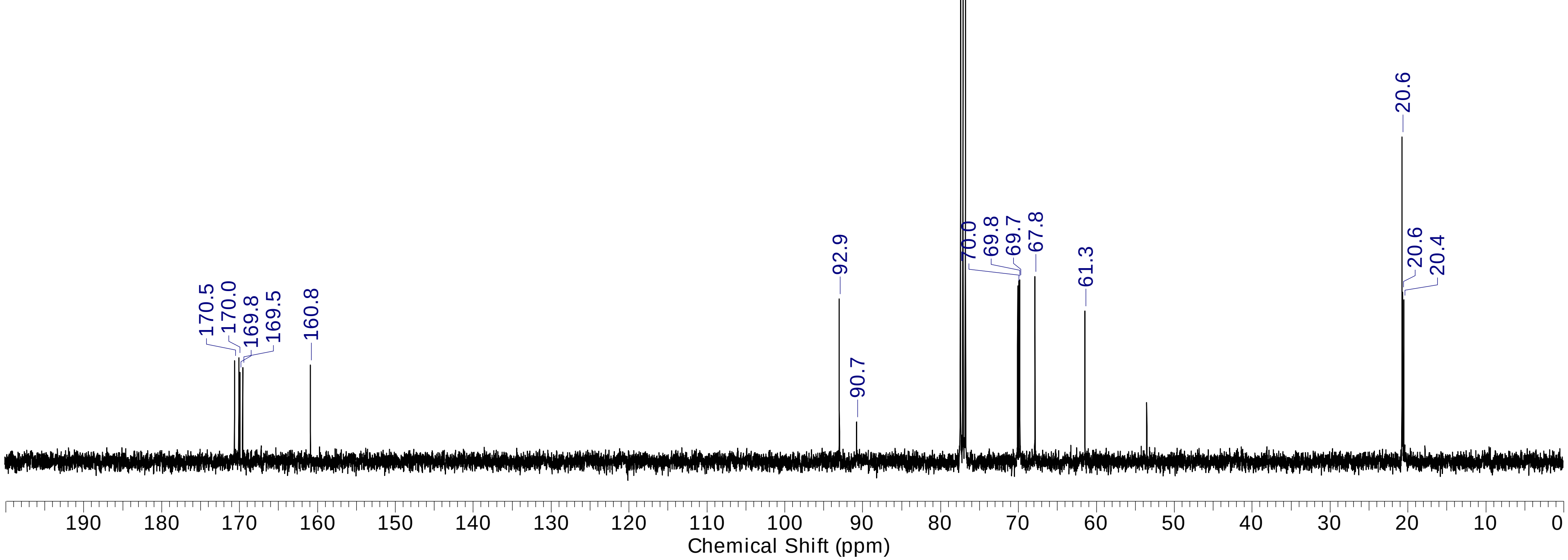

^{1}H NMR spectrum of propargyl 2,3,4,6-tetra-*O*-acetyl-β-D-glucopyranoside (6)

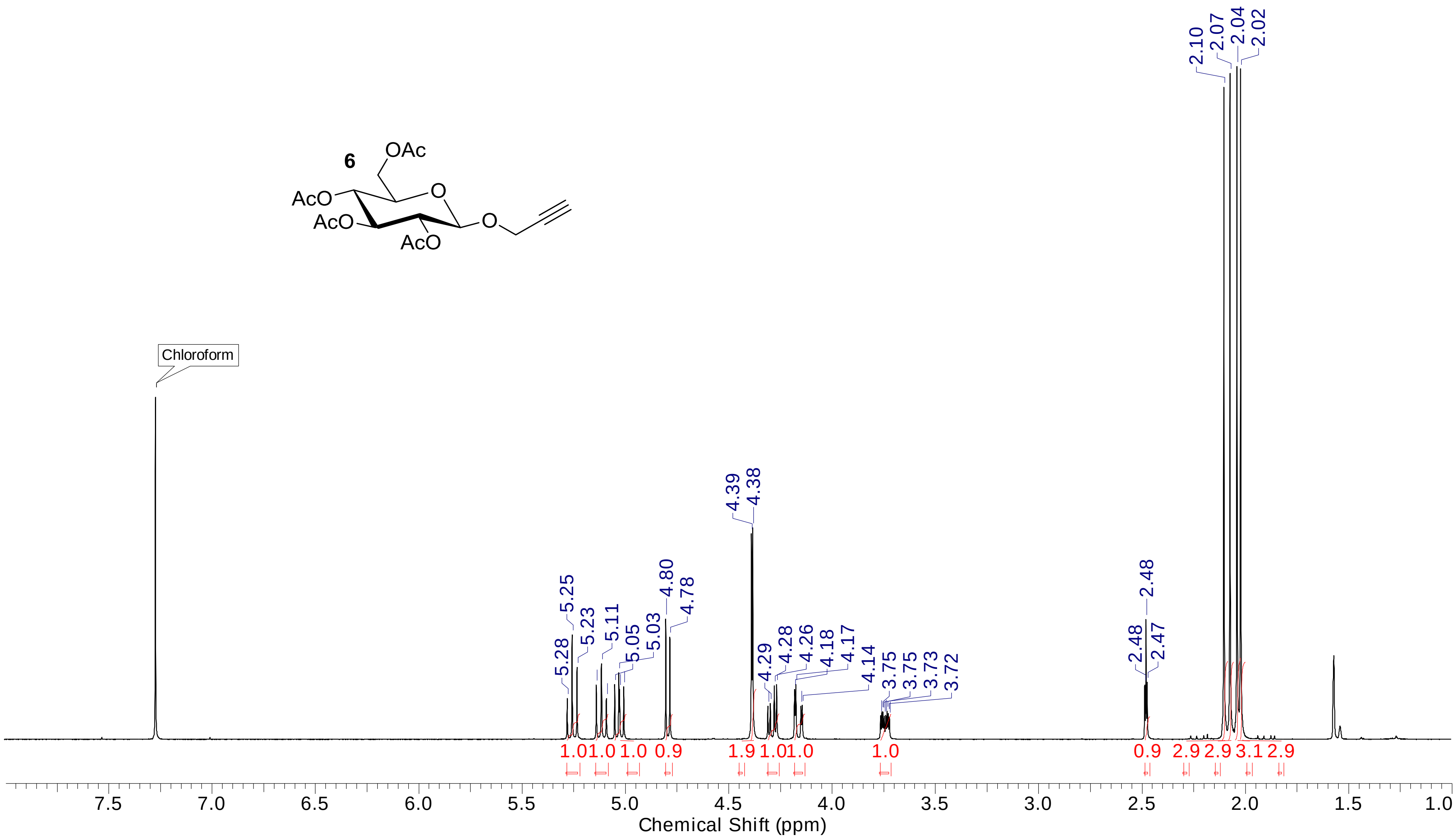

^{13}C NMR spectrum of propargyl 2,3,4,6-tetra-*O*-acetyl-β-D-glucopyranoside (6)

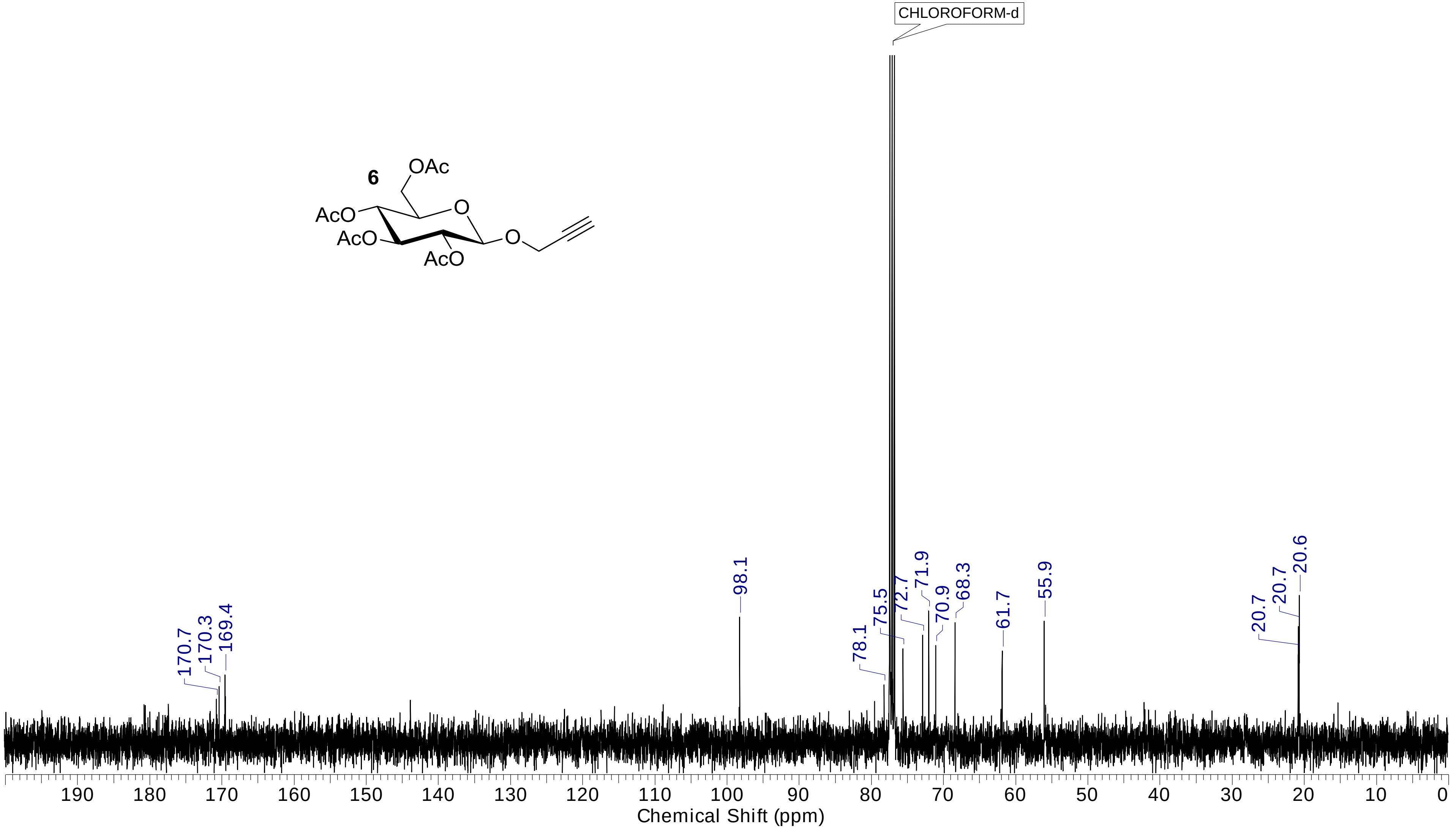

^{1}H NMR spectrum of 4-pentynyl 2,3,4,6-tetra-*O*-acetyl-β-D-glucopyranoside (7)

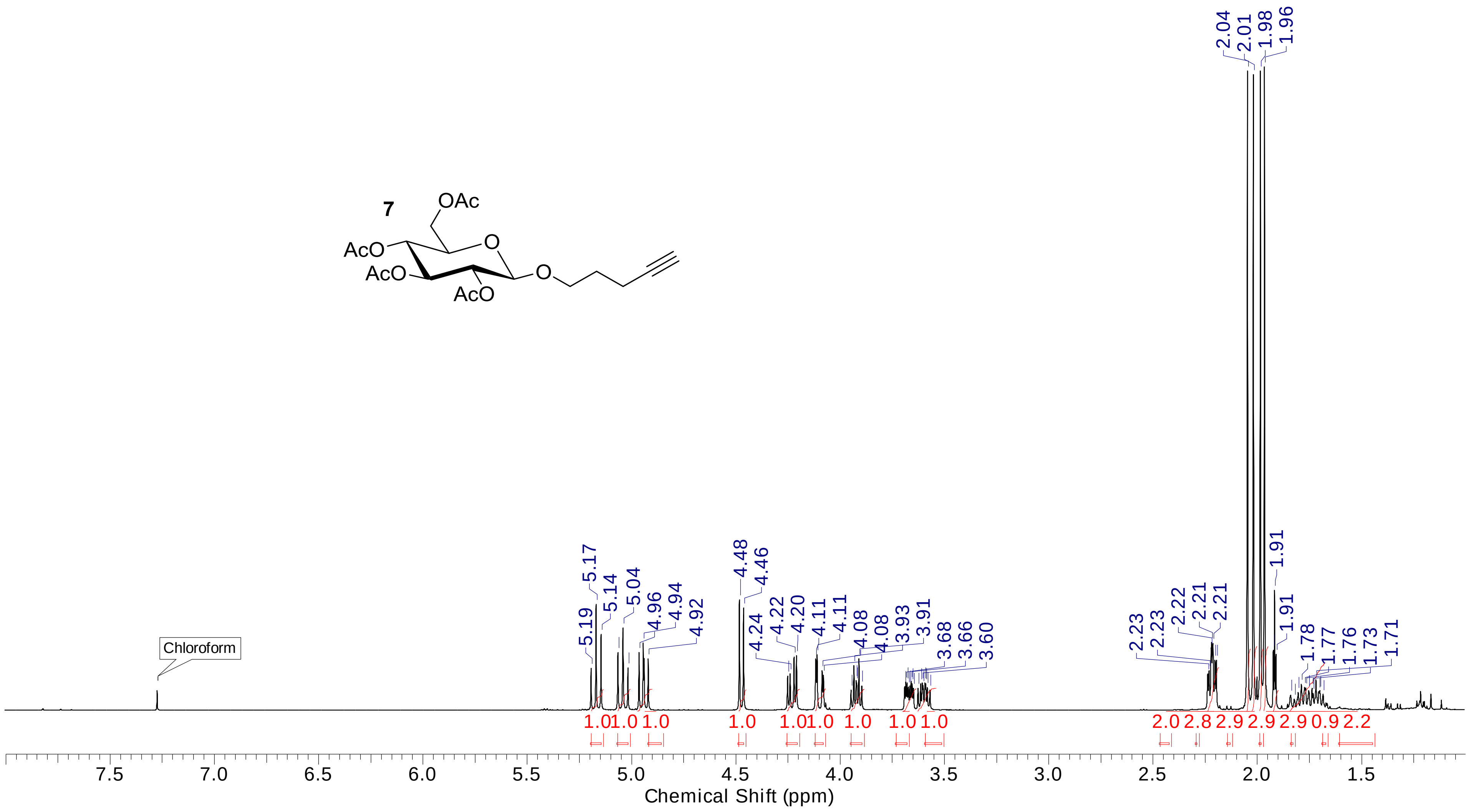

^{13}C NMR spectrum of 4-pentynyl 2,3,4,6-tetra-*O*-acetyl-β-D-glucopyranoside (7)

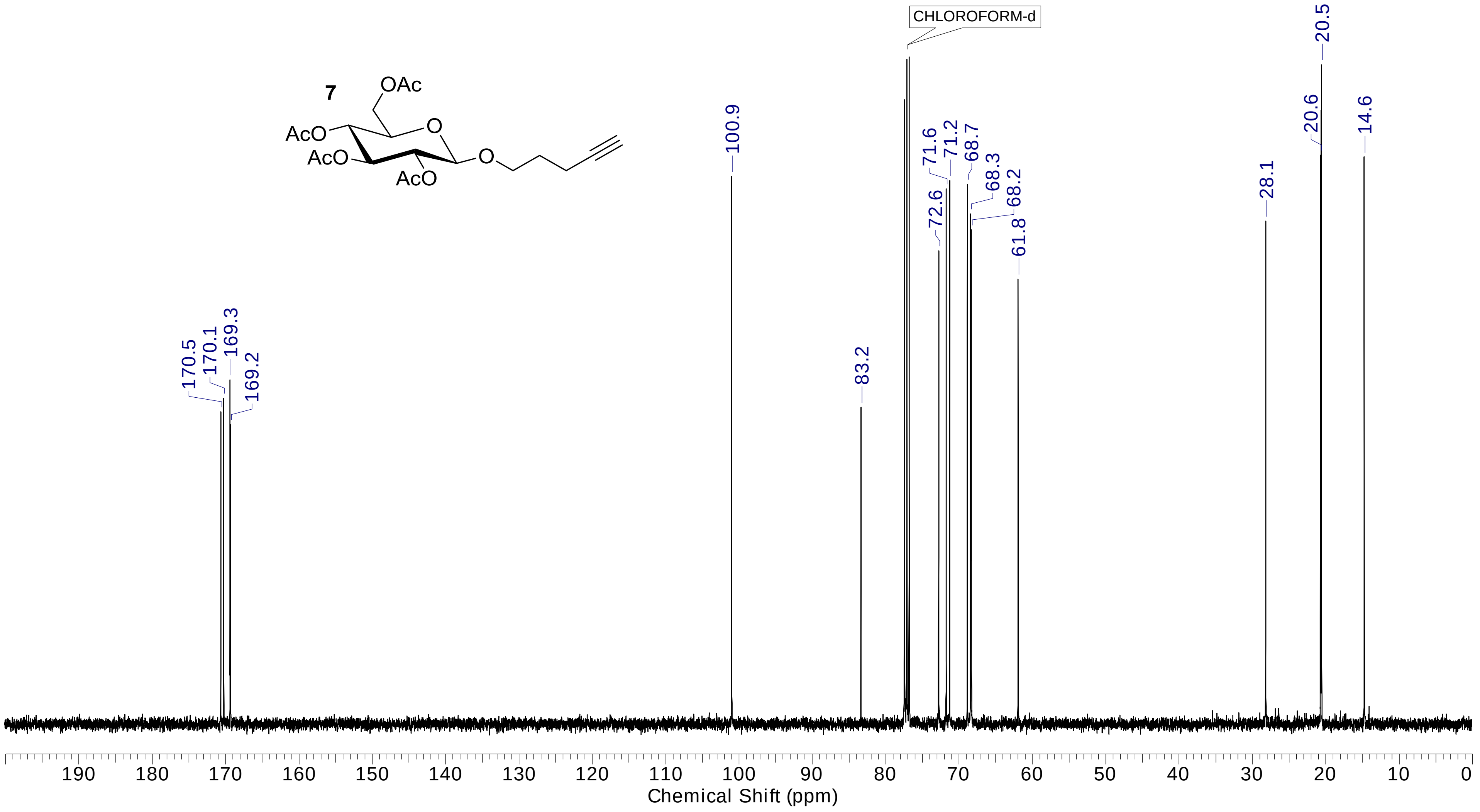

^{1}H NMR spectrum of 2,3,4,6-tetra-*O*-acetyl-β-D-glucopyranosyl azide (8)

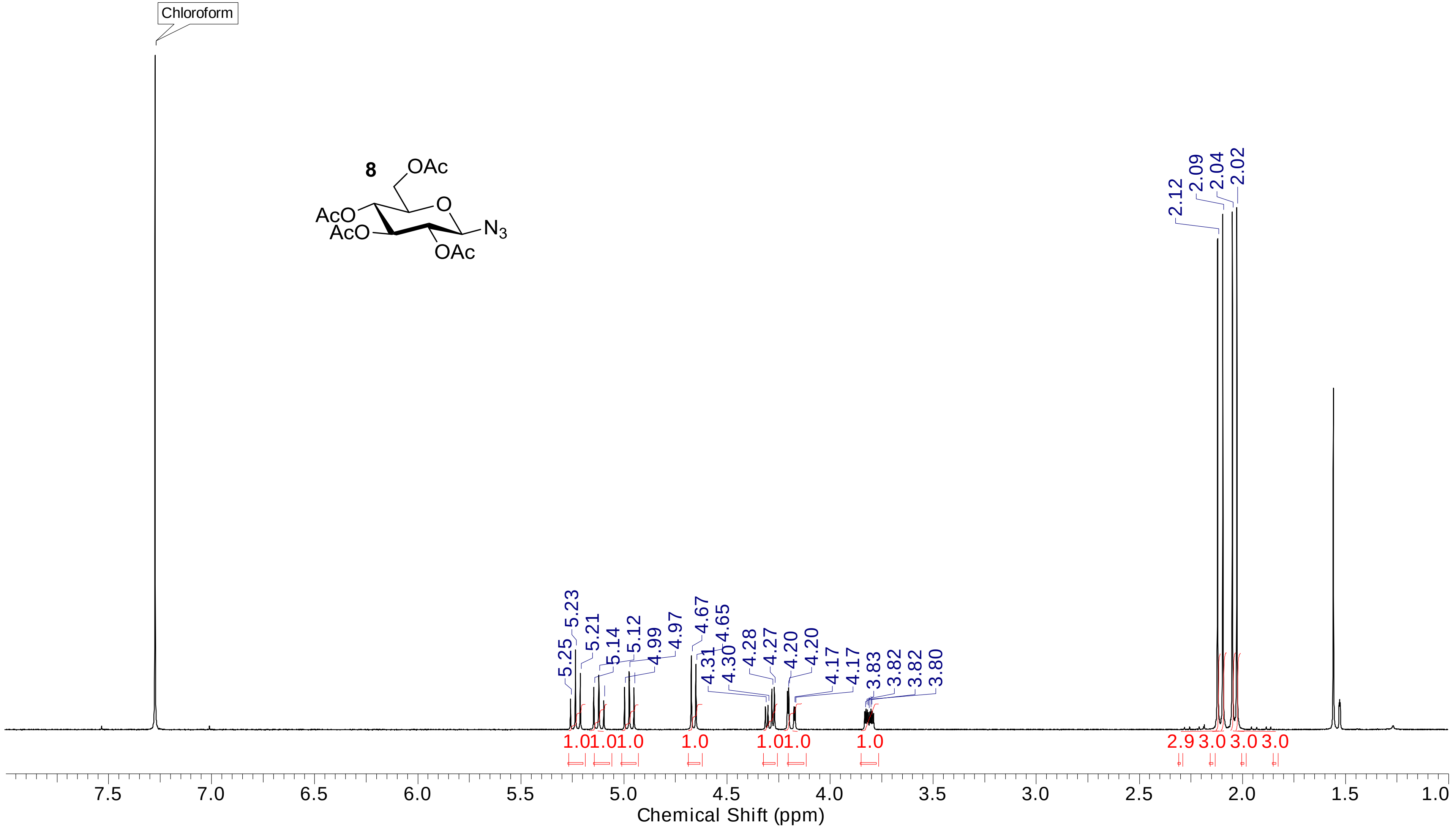

^{13}C NMR spectrum of 2,3,4,6-tetra-*O*-acetyl-β-D-glucopyranosyl azide (8)

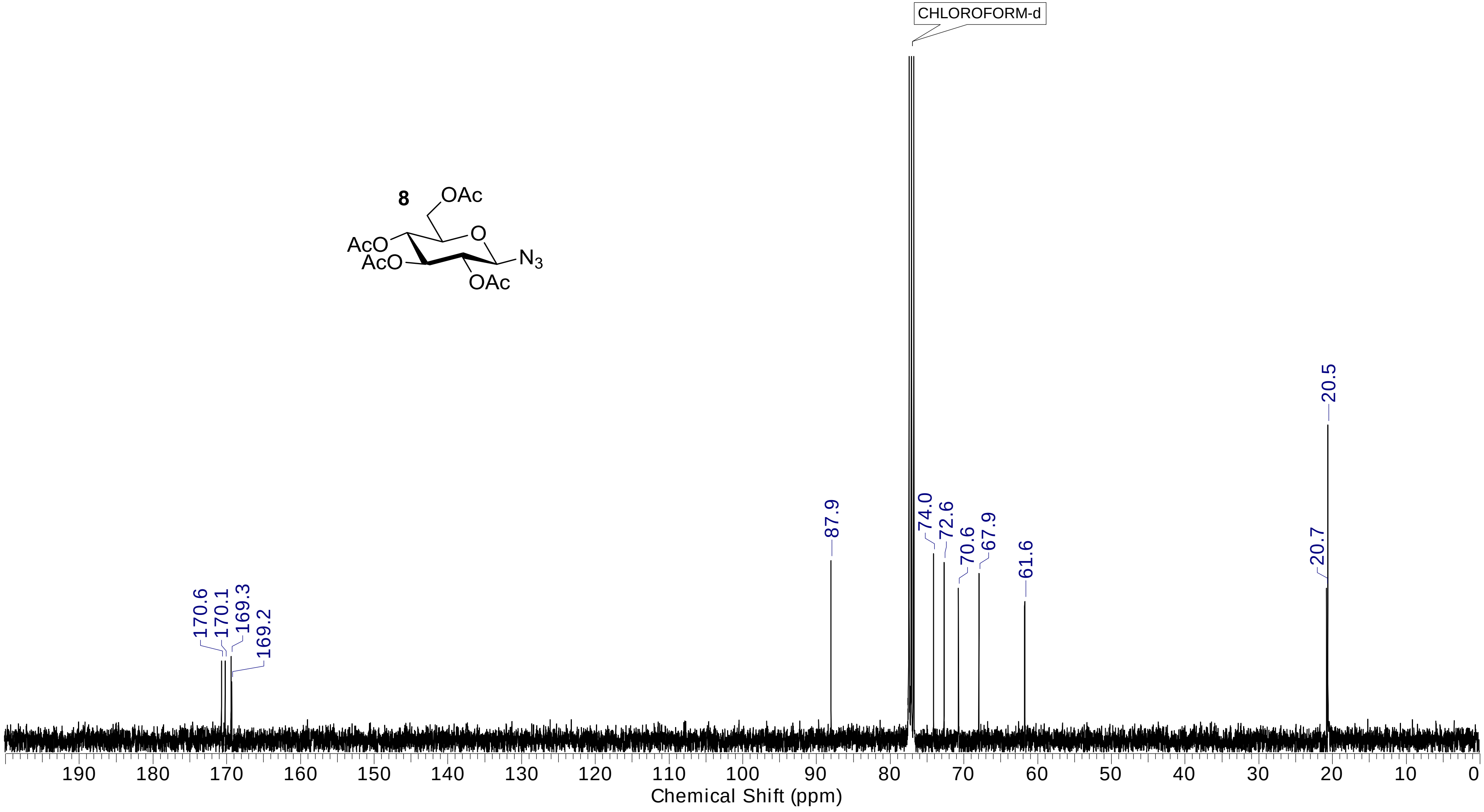

References

[1] Kartha, K. P. R.; Jennings, H. J. *J. Carbohydr. Chem.* **1990**, *9*, 777–781.

[2] Nóbrega, C.; Vázquez, J. T. *Tetrahedron: Asymmetry* **2003**, *14*, 2793–2801.

[3] Ikeda, K.; Morimoto, T.; Kakiuchi, K. *J. Org. Chem.* **2010**, *75*, 6279–6282.

[4] Pilgrim, W.; Murphy, P. V. *J. Org. Chem.* **2010**, *75*, 6747–6755.

[5] Ruchun, Y.; Haixin, D.; Yang, S.; Weihong, X.; Qiang, X.; Jun, W. *Lett. Org. Chem.* **2008**, *5*, 518–521.

[6] Gung, B. W.; Fox, R. M. *Tetrahedron* **2004**, *60*, 9405–9415.

[7] Neto, V.; Granet, R.; Krausz, P. *Tetrahedron* **2010**, *66*, 4633–4646.
