# Supplementary figures and images for: Low-budget 3D-printed equipment for continuous flow reactions
Source: Beilstein J Org Chem. 2019 Feb 26;15:558–66. doi: 10.3762/bjoc.15.50 (PMC6404462; doi:10.3762/bjoc.15.50)

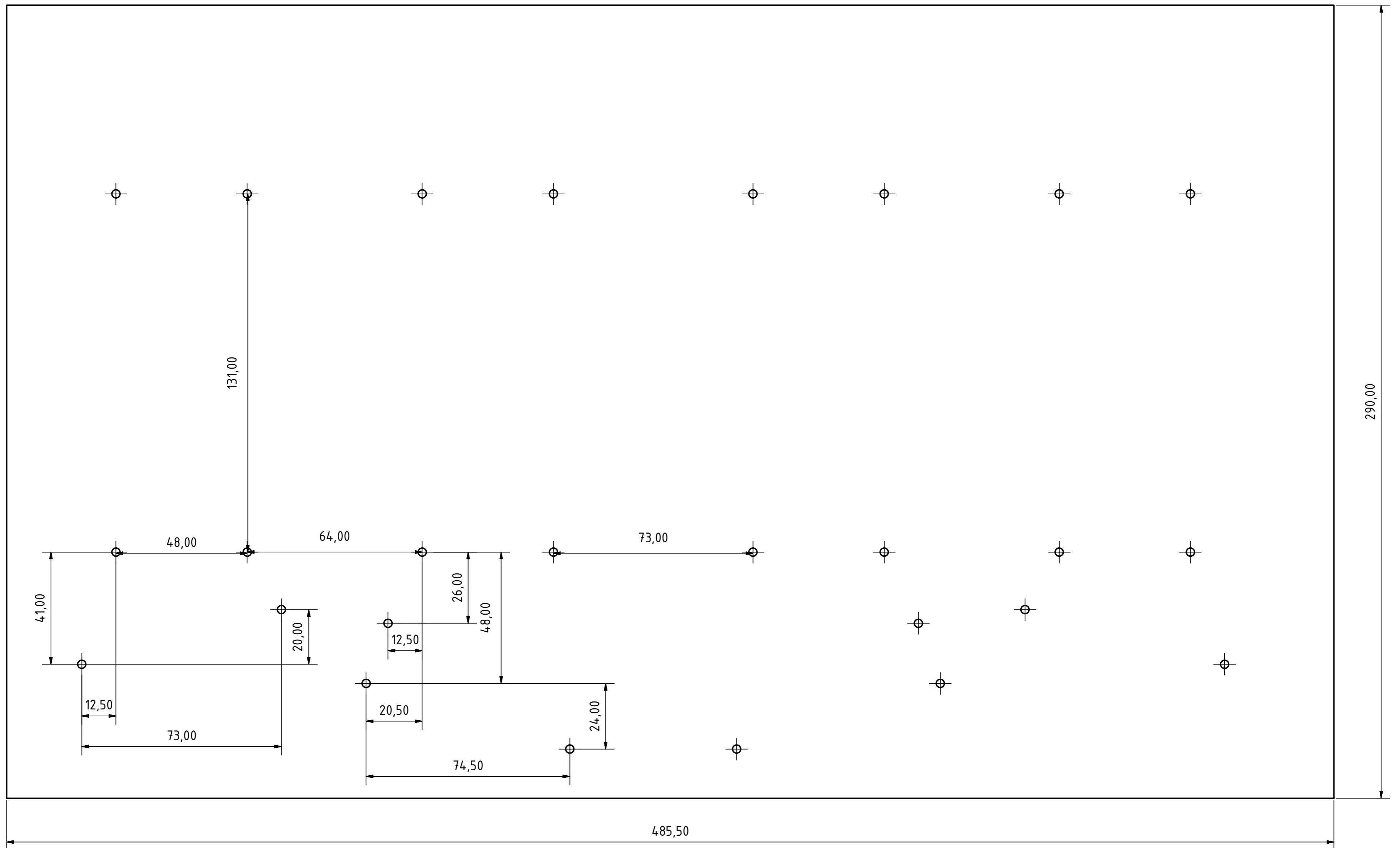

Supplement: File 2 — This zip-file includes all 3D-printed parts as stl-files for direct 3D printing, as well as stp-files for editing the 3D models, if necessary. It also contains the Arduino software code as an ino-file for controlling of the syringe pumps. [file Beilstein_J_Org_Chem-15-558-s002.zip › pump rack/Aluminium plate .pdf]
